# Effectiveness of digital health exercise interventions on muscle function and physical performance in older adults with possible, confirmed, or severe sarcopenia: a systematic review and meta-analysis

**DOI:** 10.1186/s11556-026-00409-x

**Published:** 2026-04-10

**Authors:** Ya Shi, Ying Ye, Emma Stanmore, Can Gu, Chris Todd

**Affiliations:** 1 Xiangya School of Nursing, Central South University, Changsha, China; 2 School of Health Sciences, Faculty of Biology, Medicine & Health, University of Manchester, Manchester, United Kingdom; 3 Manchester University NHS Foundation Trust, Manchester, United Kingdom

**Keywords:** Sarcopenia, Geriatrics, Older adults, Digital health, Systematic review

## Abstract

**Background:**

Digital health exercise interventions are emerging as scalable solutions for sarcopenia in older adults, but their efficacy remains unclear.

**Main body:**

This systematic review and meta-analysis evaluated their effects on muscle strength, mass, and physical performance in older adults with possible, confirmed, or severe sarcopenia. Eleven databases were searched up to 1st October 2025 for randomized and controlled clinical trials. Sixteen studies (*n* = 1,607 participants) were included. Meta-analysis revealed that, overall, digital interventions showed no significant benefit on appendicular skeletal muscle mass index (MD = 0.16 kg/m^2^, 95% CI [-0.03, 0.36]), the timed up-and-go test (SMD=-0.02, 95% CI [-0.40, 0.37]), or gait speed (SMD = 0.06, 95% CI [-0.44, 0.56]) compared to control conditions. Crucially, subgroup analyses revealed significant differential effects: combined digital health interventions (exercise plus nutrition) demonstrated superior improvements in handgrip strength (MD = 2.21 kg, 95% CI [1.33, 3.09]) and quality of life (SMD = 0.65, 95% CI [0.29, 1.01]) compared to controls. Furthermore, opposing effects on BMI were observed between exercise-focused and nutrition-focused interventions. The certainty of evidence for most outcomes was rated as low or very low.

**Conclusion:**

Standalone digital health exercise interventions confer limited benefit on core sarcopenia outcomes. In contrast, comprehensive digital interventions that combine exercise with nutritional support show significant promise for improving muscle strength, muscle mass, and quality of life, representing the most promising avenue. The current evidence base is characterized by low certainty, underscoring the need for more methodologically rigorous trials with longer-term follow-up to confirm these findings and identify the most effective intervention components and target populations.

**Trial registration:**

CRD42024516930

**Supplementary Information:**

The online version contains supplementary material available at 10.1186/s11556-026-00409-x.

## Background

Sarcopenia is a major public health concern that poses challenges in geriatric research and clinical care for ageing populations [1]. Since its initial designation by Irwin Rosenberg in 1988 [2], the definition has evolved substantially. In 2024, the steering committee of the Global Leadership Initiative in Sarcopenia (GLIS) proposed the first global conceptual definition, describing sarcopenia as an age-related, generalised skeletal muscle disease characterised by reduced muscle mass and strength with potential impairment in physical performance [3]. Nonetheless, multiple operational definitions issued by international academic organisations remain in use pending a universally accepted operational standard, including the European Working Group on Sarcopenia in Older People (EWGSOP) [4], the International Conference on Sarcopenia and Frailty Research (ICSFR) [5], the Sarcopenia Definition and Outcomes Consortium (SDOC) [6], the Asian Working Group for Sarcopenia (AWGS) [7], and the Foundation for the National Institutes of Health (FNIH) [8].

Sarcopenia is highly prevalent among older adults. A recent systematic review [9] of 19 pre-2023 studies in adults aged ≥ 65 years reported wide variation in diagnostic criteria: AWGS2 (52.63%), EWGSOP2 (21.05%), AWGS1 and EWGSOP1 (10.53% each), and FNIH (5.26%). The analysis demonstrated an age-dependent increase in prevalence from 5.32% (65–69 years) to 27% (≥ 85) irrespective of criteria [9]. Stage-specific prevalence also varies across populations: in Ireland (EWGSOP2), rates were 20.8% (possible), 8.1% (confirmed), and 5.8% (severe) [10]; in Spain (EWGSOP2), 18.8%, 3.0%, and 4.5%, respectively [11]; and in China (AWGS2), substantially higher rates of 38.5%, 18.6%, and 8.0% were observed [12]. Untreated sarcopenia accelerates physical decline and increases healthcare burdens. The condition elevates comorbidities (e.g. diabetes, cardiovascular diseases, frailty) [13–15], falls and fracture [16, 17], functional impairment [18–20], and mortality [21, 22]. It also imposes substantial socioeconomic costs, as affected older adults experience greater healthcare utilisation [23], higher hospitalisation rates [24], increased rehabilitation expenses [25], and greater catastrophic health expenditure [26]. These findings underscore the necessity of early detection, diagnosis, and management strategies.

No pharmacological agents are currently approved for sarcopenia; therefore, exercise remains the cornerstone of management [27]. Evidence-based guidelines from the ICSFR strongly recommend physical activity as a primary intervention [5]. Systematic and umbrella reviews [28–33] further support that exercise, particularly resistance training, either alone or in combination with nutritional interventions, can markedly improve muscle function and physical performance in older adults. With the expansion of digital technologies, digital health exercise interventions have gained prominence. In this review, these refer to exercise programmes delivered or supported via internet-connected platforms (e.g., mobile applications, video conferencing, wearable devices, and exergaming), enabling remote supervision and monitoring [34–36]. Such approaches facilitate home-based, scalable resistance training, promote adherence and personalised progression, and may overcome geographic and resource barriers associated with traditional supervised exercise [37–39].

Building on these advantages, multiple studies have developed digital health exercise interventions for sarcopenia prevention or treatment in older adults [40–43]. Recent systematic reviews have synthesised digital or technology-based interventions targeting sarcopenia and related muscle outcomes [44–46]. However, key limitations hinder clinical translation: prior reviews have broadly pooled heterogeneous modalities without isolating structured digital exercise programmes [44, 45]; have often focused on healthy or community-dwelling populations, limiting applicability across the full sarcopenia spectrum [45, 46]; and have insufficiently differentiated intervention structures (e.g., exercise alone vs. combined exercise and nutrition) and delivery models, potentially contributing to the substantial heterogeneity reported in previous meta-analyses [44, 46].

To address these limitations, the present review specifically focuses on digital health exercise interventions in older adults with possible, confirmed, or severe sarcopenia. We provide a structured synthesis of intervention characteristics and a comparative evaluation of different digital delivery modalities and multimodal strategies, thereby extending and refining the current evidence base.

### Aims and objectives

The overall aim of the systematic review and meta-analysis is to assess the effectiveness of digital health exercise interventions for sarcopenia prevention and treatment among older adults. The specific objectives include:

1. To systematically summarise the characteristics (e.g. exercise modalities, duration, intensity, delivery tools) of digital health exercise interventions for older adults with the three categories of sarcopenia.
2. To explore the influence of digital health exercise interventions on sarcopenic indices (muscle mass, muscle strength, and physical performance) in older adults before and after intervention.
3. To assess the comparative effectiveness of digital health exercise interventions in preventing and treating sarcopenia, as opposed to digital health nutrition interventions, digital health exercise and nutrition combined interventions, or conventional interventions.
4. To analyse which type of remote platform offers optimal benefits in older adults for sarcopenia prevention and treatment.

## Methods

### Reporting

This systematic review and meta-analysis is reported in accordance with the Preferred Reporting Items for Systematic Review and Meta-Analyses (PRISMA) 2020 statement [47]. The review methodology was preregistered on the International Prospective Register of Systematic Reviews (PROSPERO) under registration number CRD42024516930.

### Database search

Randomised controlled trials and controlled clinical trials published before 1 October 2025 were identified through 11 databases: Medline, Embase, Cochrane Central Register of Controlled Trials, Cumulated Index to Nursing and Allied Health Literature (CINAHL), Psychological Information (PsycINFO), Web of Science (WOS), Scopus, Chinese Biomedical Literature Database (CBM), Chinese National Knowledge Infrastructure (CNKI), Wan Fang Database (WANFANG), Chinese Science and Technology Periodical Database (VIP). Grey literature was searched via Google Scholar, and two trial registries (Clinicaltrials.gov and the WHO International Clinical Trials Registry Platform) were screened for unpublished trials. The search strategy, developed with a professional librarian, focused on population, intervention and research design without language or date restrictions, using expanded terms related to ‘older adults’, ‘sarcopenia’, ‘digital health’, ‘exercise’, and ‘controlled trial’. Reference lists of included studies were also examined. The initial search strategy was developed for WOS and then manually tailored to each database by the research team to account for differences in controlled vocabulary and syntax. Full search strategies for all databases are available in Supplementary Material 1.

### Eligibility criteria

The PICO (Population, Intervention, Comparators, Outcomes) principle [48] was employed to define the eligibility criteria for this review. Primary studies meeting the specified criteria were selected: (1) Older adults (≥ 60years) diagnosed with sarcopenia (possible, confirmed, or severe sarcopenia) [4, 7]; (2) Studies with any form of digital health exercise (e.g., resistance, aerobic, and balance training) lasting at least 4 weeks [49]; (3) Studies with research groups performing no digital health exercise interventions (e.g., traditional intervention without Internet devices, digital nutrition/health education/usual care) or a sham digital health exercise intervention; (4) Studies evaluating muscle strength, muscle mass, physical performance, or other measurements (e.g., body fat, nutrition, and quality of life) after intervention. Unfinished studies, qualitative studies, studies on sarcopenia concomitant with other diseases, and studies presented as case reports, reviews, conference presentations, letters, or other non-specific formats, as well as those providing inadequate data or results, were excluded.

### Review process and data extraction

Two researchers independently searched the databases using the predefined strategy. References were imported into EndNote for automated deduplication, followed by manual verification, and then uploaded to Rayyan for screening. Title, abstract, and keyword screening, as well as full-text assessment, were performed independently by both reviewers against the eligibility criteria. Data extracted included study characteristics (author, year, setting, period, sample size, age, sex), diagnostic criteria and sarcopenia category, intervention characteristics (type, delivery devices, digital platforms, content, dose), and outcomes. Any discrepancies in selection and extraction were resolved by consensus with a third researcher.

### Quality assessment

Risk of bias was evaluated using three Cochrane tools according to study design. Individually randomised controlled trials were assessed with RoB 2 across five domains (randomisation, deviations from intended interventions, missing data, outcome measurement, and selective reporting) [50]. Cluster-RCTs were appraised using the RoB 2 extension for cluster trials, incorporating cluster-level randomisation and recruitment timing [51]. Non-randomised studies of interventions were assessed with ROBINS-I, which examines seven domains spanning pre-intervention, peri-/post-intervention, and reporting biases against a hypothetical target trial [52].

The domain “bias in measurement of the outcome” was evaluated separately for objective (e.g., instrument-based physical performance, bioelectrical impedance analysis) and subjective outcomes (e.g., self-reported physical activity, quality of life), given their differing susceptibility to measurement bias. Risk levels were classified as ‘low risk’, ‘some concerns’, or ‘high risk’ (RoB 2) and ‘low’, ‘moderate’, ‘serious’, or ‘critical’ (ROBINS-I), with overall study-level judgments derived from domain ratings. Although assessment was outcome-specific in principle, a single overall judgment per study was assigned for objective and subjective outcome categories, as randomisation and intervention procedures were consistent within studies; outcomes not reported were not assessed. Results were presented using a traffic light plot (study-level domain ratings) and a summary bar chart (overall distribution of bias). Two reviewers independently performed the assessments, with inter-rater agreement quantified using Cohen’s kappa and disagreements resolved by consensus.

### Data synthesis and certainty of the evidence

A summary table described key trial characteristics, and risk-of-bias results were illustrated graphically and narratively synthesised. Meta-analyses (effect sizes, heterogeneity, subgroup and sensitivity analyses, and publication bias) were conducted using RevMan (V.5.4). Outcomes were pooled only when at least three independent studies were available. If substantial heterogeneity was observed (I² > 75%, per the Cochrane Handbook [53]) and could not be resolved through pre-specified subgroup or sensitivity analyses, the outcome was excluded from the primary meta-analysis. The certainty of evidence was appraised using the Grading of Recommendations, Assessment, Development and Evaluation (GRADE) approach and rated as high, moderate, low, or very low [54, 55].

## Results

### Study selection

**Fig. 1:**
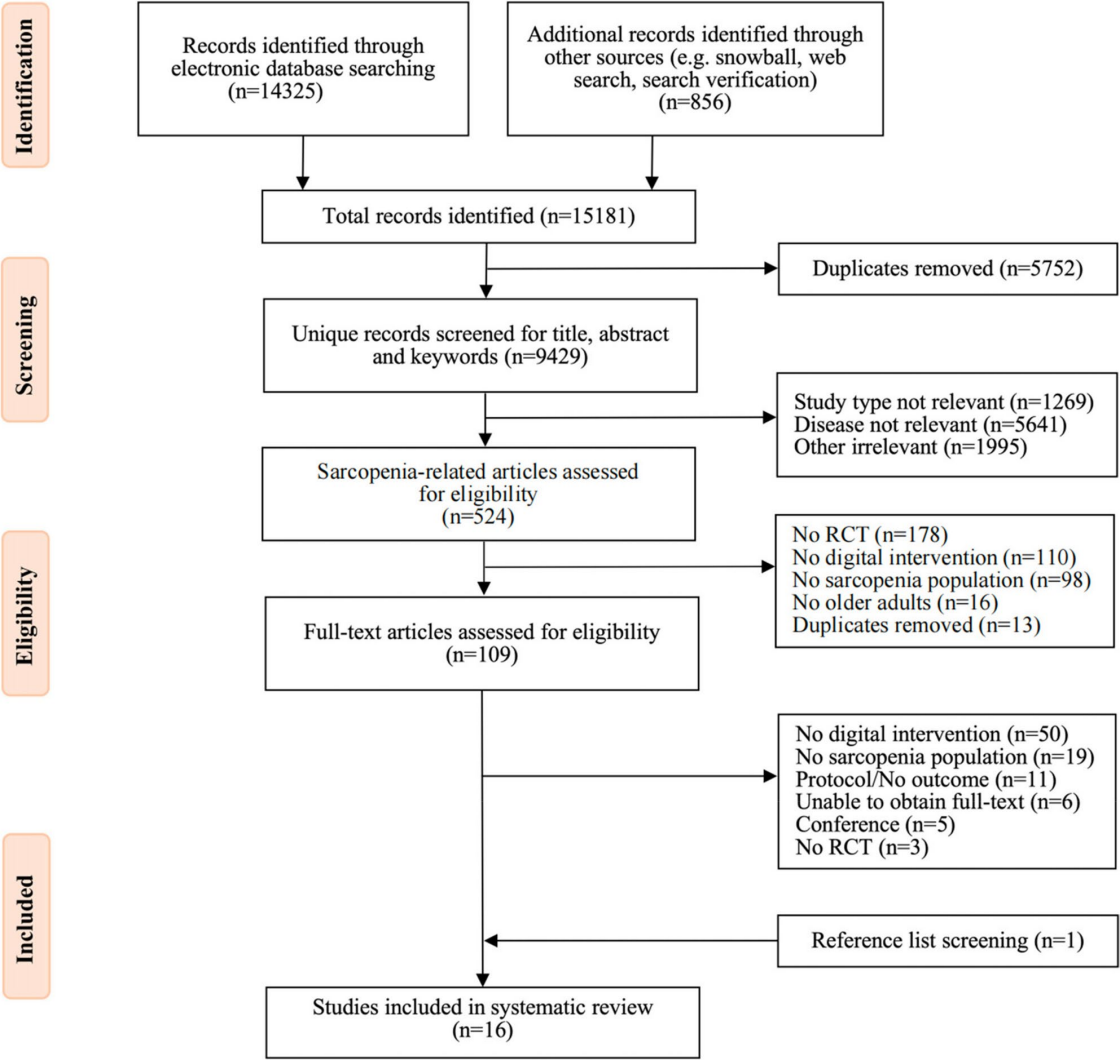
PRISMA flowchart of study selection process

A total of 14,325 records were identified through database searches, with an additional 856 records sourced from other channels. After removing 5,752 duplicates from the combined 15,181 records, 9,429 records underwent rapid screening based on title. Following the exclusion of irrelevant publications (including protocols, cross-sectional studies, and studies not related to sarcopenia), 524 sarcopenia-related records were retained for further evaluation (abstract and keywords). After further excluding studies that were not randomized controlled trials, lacked digital interventions, did not involve sarcopenia populations, or did not focus on older adults, 109 full-text articles were assessed for eligibility. Based on the inclusion and exclusion criteria, 15 records were selected. One additional record was identified from reference lists, resulting in a total of 16 studies included in the systematic review (Fig. 1). Inter-rater reliability was substantial at the title/abstract stage (*n* = 524; κ = 0.715, 95% CI 0.644–0.786; 97 inclusions, 373 exclusions, 54 disagreements) and almost perfect at the full-text stage (*n* = 109; κ = 0.821, 95% CI 0.670–0.973; 14 inclusions, 90 exclusions, 5 disagreements) [56]. The complete search terms and search strategy are provided in the Supplementary Material 1.

### Basic characteristics

The basic characteristics of the included studies are presented in Table 1. A total of 16 studies were published between 2017 and 2025, with a marked increase in publications during 2024 (*n* = 6, 37.50%) and 2025 (*n* = 5, 31.25%). Regarding study design, eight studies (50.00%) adopted an individually randomized parallel-group trial design, four (25.00%) used a cluster-randomized trial design, two (12.50%) were quasi-experimental, one (6.25%) was a preliminary randomized controlled trial, and one (6.25%) was a pilot randomized controlled trial. In terms of study location, 11 studies (68.75%) were conducted in China, two (12.50%) in Korea, two (12.50%) in the Netherlands, and one (6.25%) in Italy. With respect to the research setting, 11 studies (68.75%) were conducted in community settings, two (12.50%) in nursing homes, and three (18.75%) in hospital-to-home settings.

**Table 1:** Basic characteristics of the included studies

Code	Refs	Year	Design	Site	Diagnostic Criteria	Sarcopenia category	Intervention Period	Follow-up	*N* (Complete/Recruit)
Y1	Hong et al. [43]	2017	RCT	Korea Community	Kim et al. [57]	Not mentioned (prevention)	12-week	None	23/26
Y2	van den Helder et al. [58]	2020	Cluster RCT	Netherlands Community	Not mentioned	Probable + Confirmed sarcopenia (in a subset of participants)	24-week	24-week	168/184/224
Y3	van den Helder et al. [59]	2021	Cluster RCT	Netherlands Community	Not mentioned	Probable + Confirmed sarcopenia (in a subset of participants)	24-week	24-week	167/181/212
Y4	Wang et al. [42]	2022	Cluster RCT	China Community	EWGSOP2 + AWGS2	Confirmed sarcopenia	12-week	None	201/234
Y5	Zhang et al. [60]	2023	RCT	China Hospital-to-home	Not mentioned	Confirmed sarcopenia	24-week	None	144/144
Y6	Tuan et al. [61]	2024	RCT	China Nursing home	Not mentioned	Probable + Confirmed + Severe sarcopenia (in a subset of participants)	12-week	None	55/60
Y7	Bonato et al. [62]	2024	RCT	Italy Hospital-to-home	EWGSOP2 + Gonzalez et al. [63]	Confirmed sarcopenia	48-week	None	129/157
Y8	Ji et al. [64]	2024	RCT	China Community	AWGS2	Confirmed sarcopenia	12-week	None	80/80
Y9	He et al. [65]	2024	RCT	China Community	AWGS2	Confirmed sarcopenia	12-week	None	70/75
Y10	Ho et al. [66]	2024	Cluster RCT	China Community	AWGS2	Not mentioned (prevention)	8-week	None	58/58
Y11	Guo et al. [67]	2024	Quasi-experimental (non-concurrent control)	China Community	AWGS2	Pre-sarcopenia + Confirmed + Severe sarcopenia	12-week	None	44/46
Y12	Zhang et al. [68]	2025	RCT	China Hospital-to-home	AWGS2	Confirmed sarcopenia	4-week	None	51/58
Y13	An et al. [69]	2025	Preliminary RCT	Korea Community	AWGS2	Confirmed sarcopenia	4-week	None	30/30
Y14	Wei et al. [70]	2025	RCT	China Community	AWGS2	Confirmed sarcopenia	12-week	None	76/93
Y15	Wu et al. [71]	2025	Quasi-experimental	China Community	AWGS2	Probable sarcopenia	12-week	None	80/80
Y16	Liu et al. [72]	2025	Pilot RCT	China Nursing home	AWGS2	Probable sarcopenia	12-week	12-week	24/24/ 30

“?” means data not reported. The letters a, b, and c respectively represent the different digital intervention groups in the included studies

Detailed intervention characteristics for each included study are provided in Supplementary Material 2

Concerning the diagnostic criteria for sarcopenia, 10 studies (62.50%) explicitly adopted the criteria established by the AWGS2, two (12.50%) used the EWGSOP2 criteria, and two (12.50%) applied criteria set by individual studies. The remaining four studies (25.00%) did not specify the diagnostic criteria. Eight studies (50.00%) focused on participants with confirmed sarcopenia, two (12.50%) on probable sarcopenia, four (25.00%) included two or more sarcopenia categories, and two (12.50%) focused on sarcopenia prevention without specifying the category. Altogether, 1,607 older adults were enrolled across the 16 studies, of whom 1,430 completed the intervention phase, yielding an overall completion rate of 88.99%. Fourteen studies reported the gender distribution, among which nine had a higher proportion of female participants. In addition, 13 studies reported the mean age of participants in the intervention groups, ranging from 68.18 ± 3.93 to 82.20 ± 5.60 years.

### Intervention characteristics

#### General information

As shown in Table 1 and Supplementary Material 2, the intervention duration across the 16 included studies ranged from 4 to 48 weeks. Among them, nine studies (56.25%) implemented 12-week interventions, three (18.75%) lasted 24 weeks, two (12.50%) lasted 4 weeks, one (6.25%) lasted 8 weeks, and one (6.25%) extended to 48 weeks. Only three studies (18.75%) conducted a follow-up assessment after the intervention period. Across the 16 studies, a total of 22 digital intervention groups and 16 traditional offline control groups were established. Among the digital intervention groups, 14 (63.64%) involved exercise interventions, 5 (22.73%) combined exercise and nutrition, 2 (9.09%) combined exercise and health education, and 1 (4.55%) involved nutrition intervention alone. The 16 offline control groups included 5 (31.25%) offline exercise groups, 1 (6.25%) combined exercise and nutrition group, 5 (31.25%) offline health education groups, and 5 (31.25%) groups maintaining their original lifestyle.

#### Digital intervention tools

The digital intervention tools used in the included studies were extracted (Supplementary Material 2) and summarized (Table 2). A total of nine main categories of digital tools were identified. The most frequently used were video conferencing or telecommunication platforms (*n* = 5), followed by mobile or tablet applications (*n* = 3), wearable devices (*n* = 2), web-based platforms (*n* = 2), and integrated systems combining applications, wearables, and online monitoring (*n* = 2). Other tools included exergames (*n* = 2), AI-based motion analysis systems (*n* = 2), a mixed-reality platform (*n* = 1), and a social-media platform (*n* = 1). These tools were primarily used to deliver, monitor, and evaluate exercise performance, as well as to provide feedback, reminders, and adherence support.

**Table 2:**
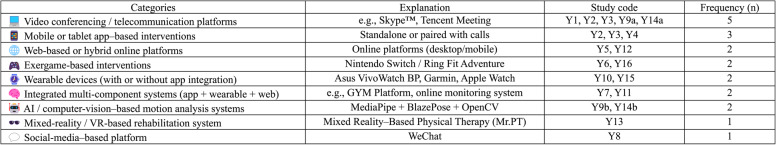
Classification and frequency of digital intervention tools used in the included studies [73]

Y1–Y16 correspond to the study codes listed in Table 1. Both studies Y9 and Y14 included two digital exercise intervention groups, which were denoted as groups a and b, respectively

#### Exercise guidelines, types, duration and intensity

The characteristics of all digital exercise interventions are summarized in Table 3. Among the 16 studies, 12 (75.00%) explicitly reported basing their exercise design on established guidelines. The most frequently cited were the American College of Sports Medicine (ACSM) guidelines (*n* = 7, 43.75%), followed by the World Health Organization (WHO) physical activity guidelines (*n* = 3, 18.75%). In addition, 6 studies (37.50%) referred to national or institutional guidelines for exercise prescription. The remaining 4 studies (25.00%) did not specify guidelines but developed their protocols through expert consultation or pilot testing to ensure safety and appropriate progression.

**Table 3:**
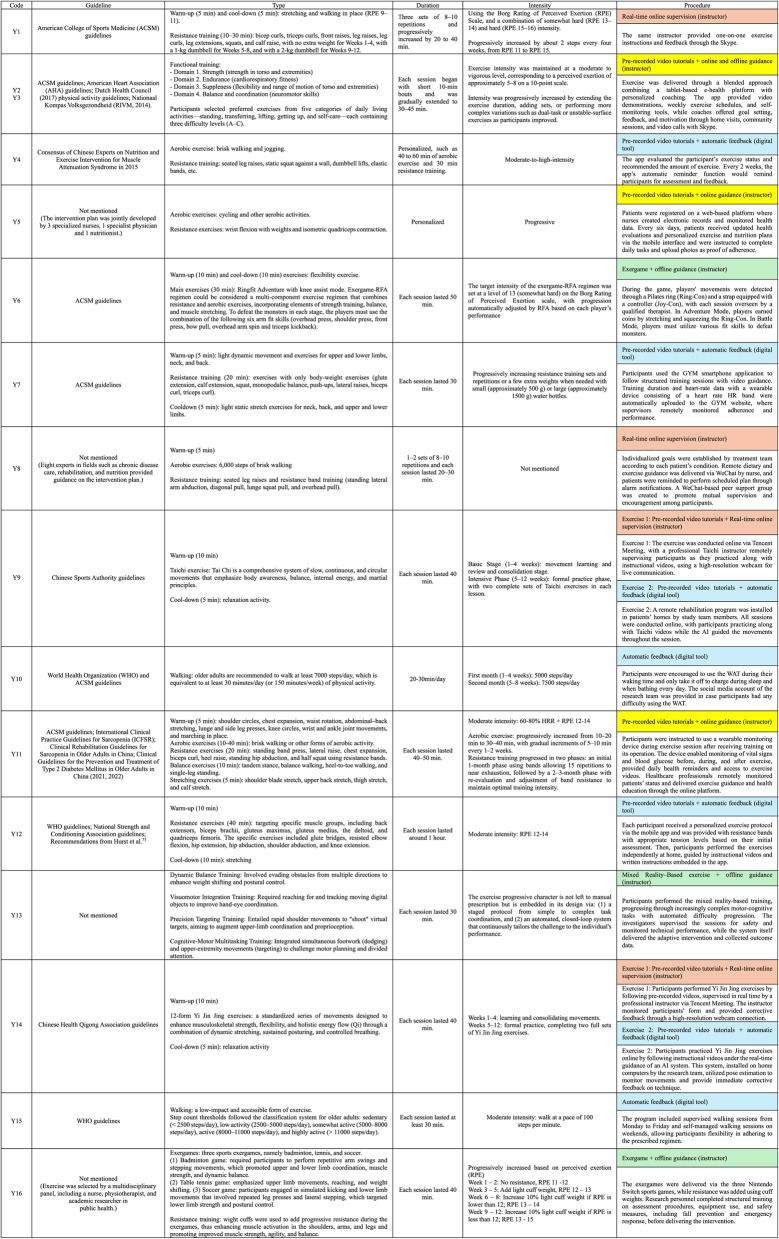
Digital exercise intervention characteristics of the included studies [73]

Y1–Y16 correspond to the study codes listed in Table 1. The same color represents the same categories of digital implementation procedures. Y12: Recommendations from Hurst et al. [73]

Regarding exercise types, 14 studies (87.50%) implemented multicomponent training, most frequently combining resistance training (*n* = 9, 56.25%), aerobic exercise (*n* = 6, 37.50%), and stretching/flexibility training (*n* = 5, 31.25%). Functional training was included in three studies (18.75%), balance training in two (12.50%), and traditional Chinese exercises (e.g., Tai Chi, Yi Jin Jing) in two (12.50%). Only two studies (12.50%) employed aerobic-only programs, such as walking. Exercise sessions generally lasted 20–60 min and were conducted 2–5 times per week in 14 studies (87.50%). Two studies (12.50%) did not report exercise intensity, whereas 14 studies (87.50%) explicitly stated it, most of which prescribed moderate-to-high intensity training based on progressive overload principles.

#### Digital intervention procedures

All included studies employed digital tools to deliver, monitor, or supervise exercise programs. Participants in the digital intervention groups followed four main implementation procedures: ① Real-time online supervision with instructors (*n* = 4); ② Pre-recorded video tutorials combined with online or offline instructor guidance (*n* = 4); ③ Exergame or mixed-reality–based exercise combined with offline guidance (*n* = 3); ④ Self-administered training supported by digital tools with or without pre-recorded video tutorials (*n* = 7). These approaches varied in the level of professional involvement and system automation, ranging from fully supervised real-time online sessions to completely self-managed digital exercise guided solely by automated feedback mechanisms, as shown in Table 3.

### Outcome characteristics

The common outcome indicators (reported in $\:\ge\:$2 studies) for sarcopenia interventions and their effects are summarized in Table 4. These indicators were identified and categorized into seven domains, with their frequencies of use across the 16 studies as follows: (i) Muscle strength: handgrip strength (*n* = 11) and arm curls (*n* = 2); (ii) Muscle mass: appendicular skeletal muscle index (*n* = 9), total-body skeletal muscle mass (*n* = 4), and appendicular lean mass (*n* = 3); (iii) Physical performance: gait speed (*n* = 7), timed up-and-go test (*n* = 6), sit-to-stand test (*n* = 4), short physical performance battery (*n* = 3), 6-minute walk test (*n* = 2), chair stand test (*n* = 2), and balance test (*n* = 2); (iv) Physical function: quality of life (*n* = 6) and activities of daily living (*n* = 3); (v) Body composition: body fat percentage (*n* = 3); (vi) Anthropometry: body mass index (*n* = 3); (vii) Nutritional intake: protein intake (*n* = 3) and energy intake (*n* = 2). A detailed summary of the measurement tools and intervention effects for each indicator is provided in Table 4.

**Table 4:**
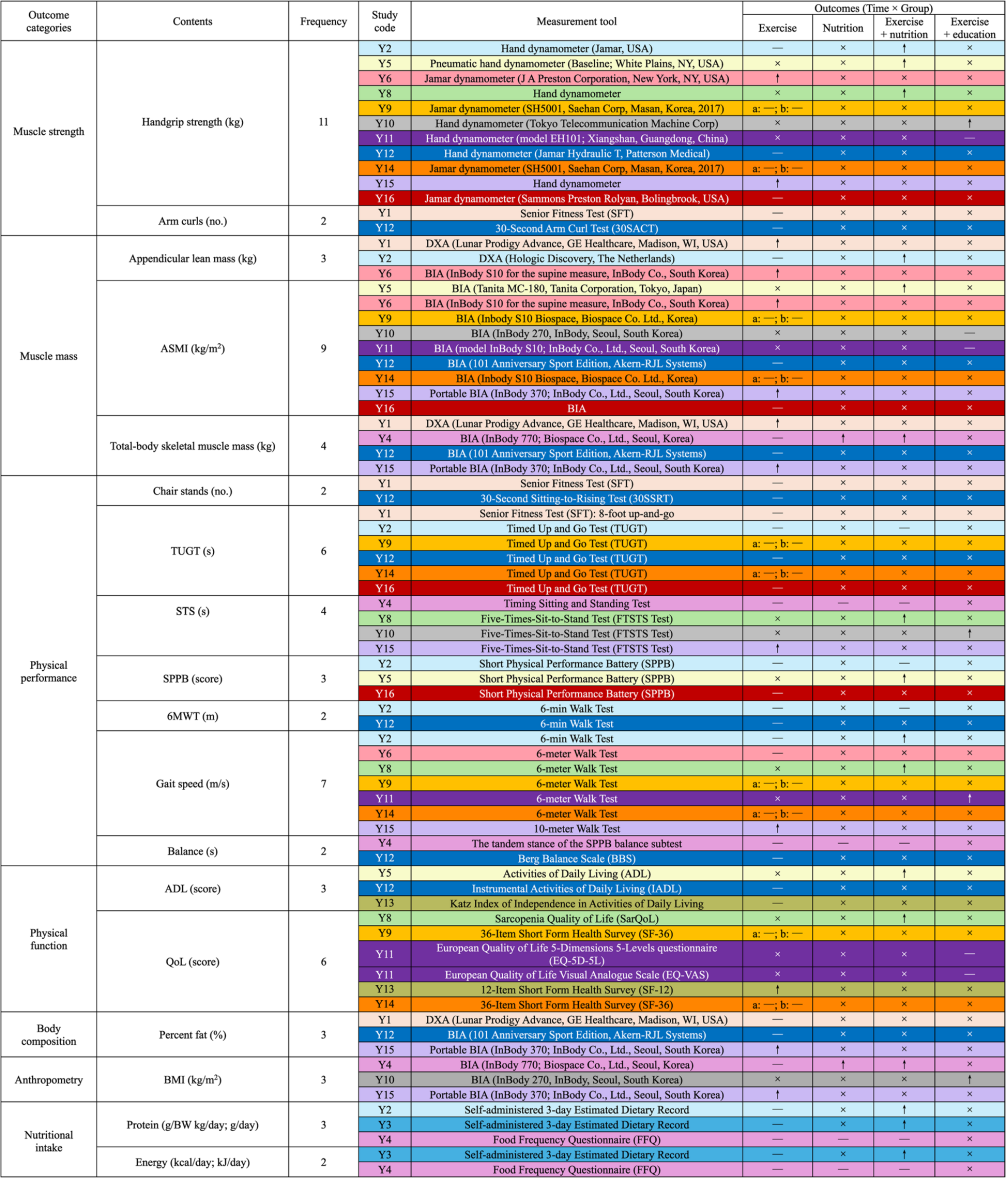
Outcome characteristics of the included studies

Y1–Y16 correspond to the study codes listed in Table 1. The same color represents the results from the same study. DXA means a dual-energy X-ray absorptiometry device. BIA means a bioelectrical impedance analyzer. x means not applicable. ↑ indicates a significant improvement in the indicator values after the digital intervention compared with the control group. — indicates no significant difference in the indicator values after the digital intervention compared with the control group

### Risk of bias assessment

**Fig. 2:**
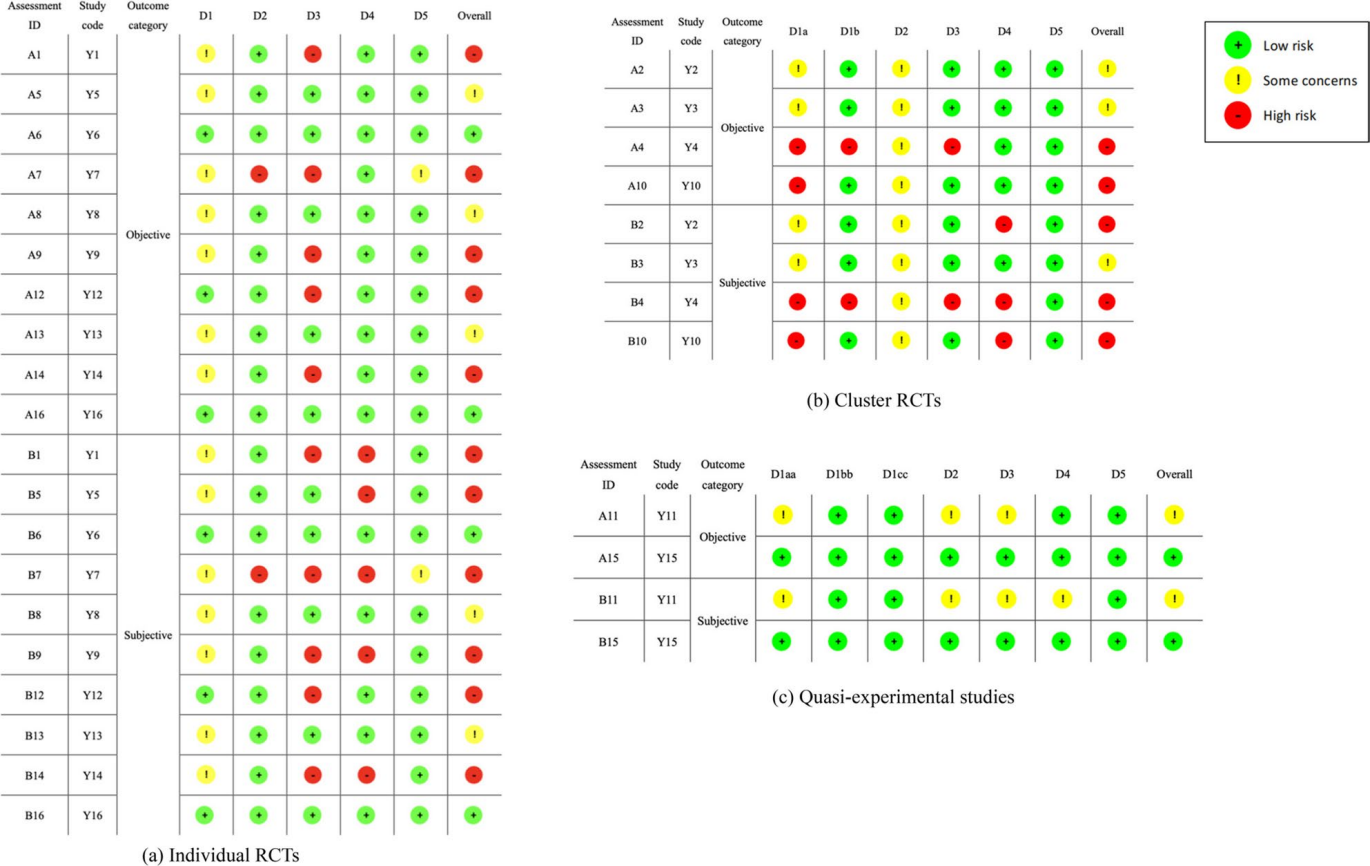
Risk of bias for individual studies. Note: Y1–Y16 correspond to the study codes listed in Table 1. D1 - bias arising from the randomisation process; D1a - bias from randomization process for the clusters; D1b - bias from randomization process for the individuals; D1aa - bias due to confounding; D1bb - bias in classification of interventions; D1cc - bias in selection of participants into the study (or into the analysis); D2 - bias due to deviations from intended interventions; D3 - bias due to missing outcome data; D4 - bias in measurement of the outcome; D5 - bias in selection of the reported result.

**Fig. 3:**
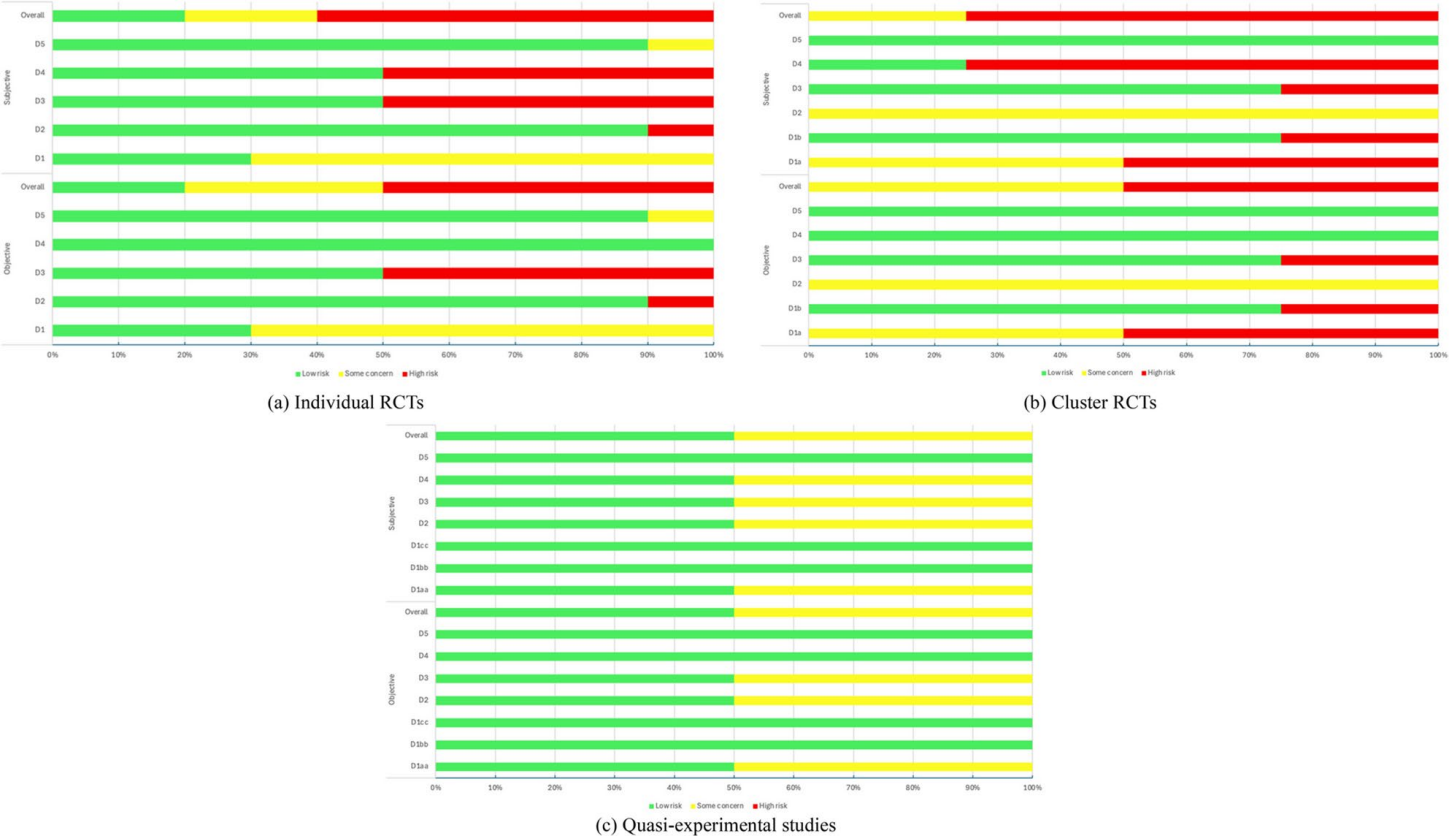
Overall summary of risk of bias. Note: D1 - bias arising from the randomisation process; D1a - bias from randomization process for the clusters; D1b - bias from randomization process for the individuals; D1aa - bias due to confounding; D1bb - bias in classification of interventions; D1cc - bias in selection of participants into the study (or into the analysis); D2 - bias due to deviations from intended interventions; D3 - bias due to missing outcome data; D4 - bias in measurement of the outcome; D5 - bias in selection of the reported result

Figure 2 presents the risk-of-bias assessment for each included study, and Fig. 3 provides the overall summary. In the RoB 2 and ROBINS-I frameworks, D1 represents pre-intervention bias, D2 represents bias arising during the intervention period, and D3–D5 represent post-intervention biases.

#### Pre-intervention bias (D1)

For the ten individually randomized trials, three studies (30%) were judged as low risk and seven studies (70%) as some concerns regarding bias arising from the randomization process (D1). Among the four cluster-randomized trials, two studies (50%) were rated as some concerns and two (50%) as high risk for the cluster-level randomization process (D1a), whereas three studies (75%) were rated as low risk and one (25%) as high risk for the individual-level randomization timing and procedures (D1b). Regarding the two quasi-experimental studies, one study (50%) was rated as low risk and one (50%) as some concerns for bias due to confounding (D1aa). Both studies were assessed as low risk for bias in classification of interventions (D1bb) and selection of participants into the study or analysis (D1cc).

#### Bias during intervention (D2)

Across all 16 studies, in the domain of deviations from intended interventions (D2), ten studies (62.5%) were judged as low risk, five (31.25%) as some concerns, and one (6.25%) as high risk.

#### Post-intervention bias (D3–D5)

For missing outcome data (D3), nine studies (56.25%) were rated low risk, one (6.25%) some concerns, and six (37.5%) high risk. For outcome measurement bias (D4), all studies (100%) were judged low risk for objective outcomes. In contrast, for subjective outcomes, seven studies (43.75%) were rated low risk, one (6.25%) some concerns, and eight (50%) high risk. Regarding selective reporting (D5), 15 studies (93.75%) were judged low risk and one (6.25%) as some concerns.

### Overall risk of bias

Based on the study-level overall judgment, for objective outcomes, three studies (18.75%) were judged as low risk, six (37.5%) as some concerns, and seven (43.75%) as high risk. For subjective outcomes, three studies (18.75%) were rated low risk, four (25%) some concerns, and nine (56.25%) high risk.

The inter-rater reliability for the overall risk-of-bias judgment was substantial for objective outcomes (κ = 0.62) and moderate for subjective outcomes (κ = 0.60), indicating an acceptable level of agreement between reviewers.

### Meta-analysis results

**Fig. 4:**
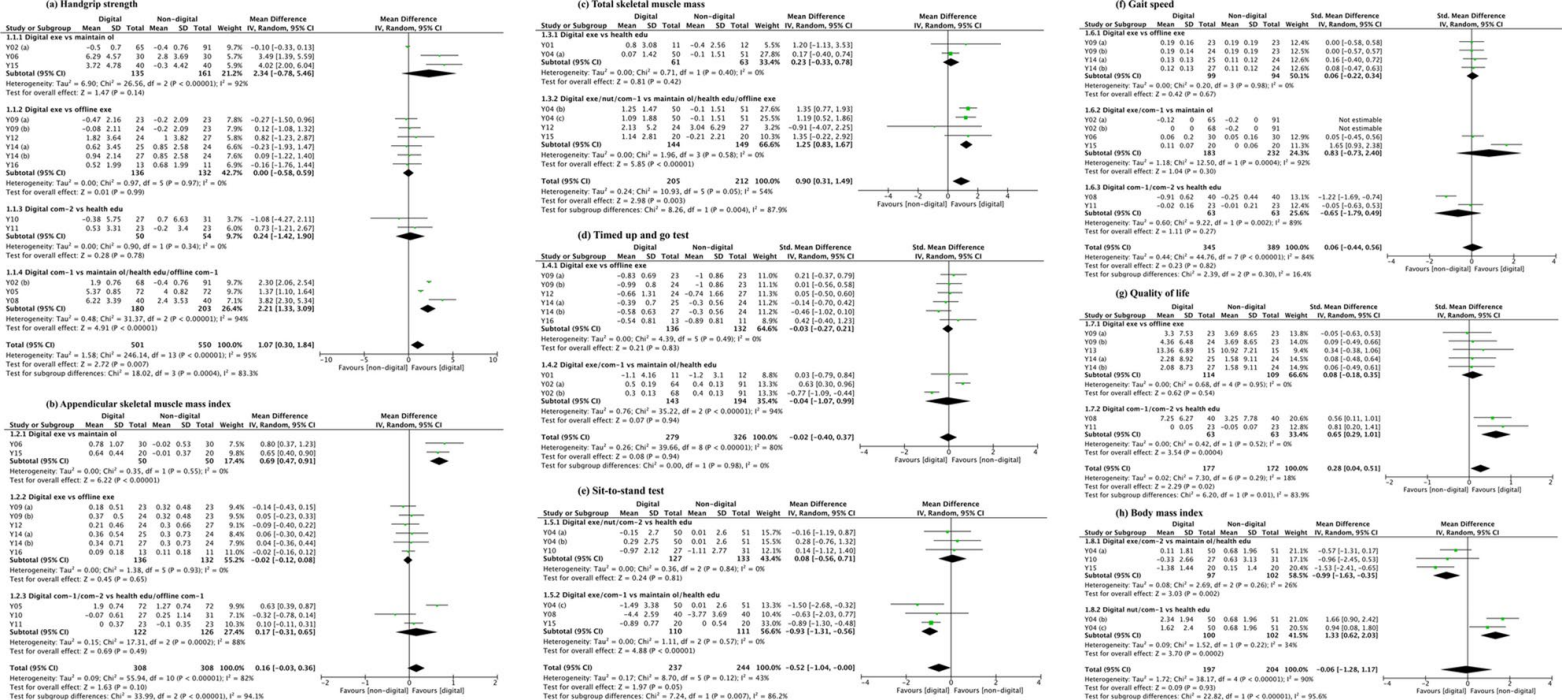
Forest plots for sarcopenia outcomes: digital versus non-digital interventions. Note: Y01–Y16 correspond to the study codes listed in Table 1. Digital exe — Digital health exercise; Digital nut — Digital nutrition; Digital com-1 — Digital health exercise + nutrition; Digital com-2 — Digital health exercise + education; Maintain ol — Maintain original lifestyle; Health edu — Health education; Offline exe — Offline exercise; Offline com-1 —Offline exercise + nutrition. Slashes indicate subgroups that pool multiple comparator conditions.

Following our pre-specified criteria, of the 18 extracted outcome measures across seven domains (as presented in Table 4), ten were excluded from the meta-analysis. The reasons for exclusion were either an insufficient number of available studies (fewer than three), or the presence of substantial and unexplained statistical heterogeneity (I² > 75%) that could not be resolved. Consequently, only eight indicators were ultimately included in the meta-analysis: handgrip strength, appendicular skeletal muscle mass index, total skeletal muscle mass, timed up-and-go test, sit-to-stand test, gait speed, quality of life, and body mass index. The results of the meta-analysis for these eight indicators are presented in Fig. 4 (a-h).

The meta-analyses revealed that the effectiveness of digital interventions was highly dependent on both the outcome measure and the specific intervention type compared to the control condition. The overall effects, heterogeneity, and results of subgroup and sensitivity analyses for all eight outcome indicators are summarized in Table 5. Due to space constraints, a detailed presentation of the results for these eight outcomes, including effect sizes, heterogeneity, subgroup analysis, and sensitivity analysis, is provided in Supplementary Material 3.

1. Handgrip strength

**Table 5:** Outcome characteristics of the included studies

Outcome indicator	Overall Effect			Subgroup Differences		Key Subgroup Findings	Key Sensitivity Findings
	MD/SMD (95% CI)	*p*-value	I² (%)	*p*-value	I² (%)		
Handgrip strength	1.07 (0.30, 1.84)	0.007	95	0.0004	83.3	Significant benefit for “Digital health exercise plus nutrition vs maintain original lifestyle/health education/offline exercise plus nutrition”.	Y02(a/b) were key heterogeneity sources.
Appendicular skeletal muscle mass index	0.16 (−0.03, 0.36)	0.10	82	< 0.00001	94.1	The improvement following “digital health exercise” was significant compared to “maintain original lifestyle” and comparable to “offline exercise”.	Y05 was a major source of heterogeneity.
Total skeletal muscle mass	0.90 (0.31, 1.49)	0.003	54	0.004	87.9	Significant benefit for “Digital health exercise/nutrition/health exercise plus nutrition vs maintain original lifestyle/health education/offline exercise”.	Robust after removing Y04b.
Timed up and go test	−0.02 (−0.40, 0.37)	0.94	80	0.98	0	The improvement following “digital health exercise” was comparable to “offline exercise”. No significant subgroup differences.	Y02(b) was a key outlier driving heterogeneity.
Sit-to-stand test	−0.52 (−1.04, 0.00)	0.05	43	0.007	86.2	Significant benefit for “Digital health exercise/health exercise plus nutrition vs maintain original lifestyle/health education”.	Effect strengthened after removing Y04b.
Gait speed	0.06 (−0.44, 0.56)	0.82	84	0.30	16.4	The improvement following “digital health exercise” was comparable to “offline exercise”. No significant subgroup differences.	Y08 and Y15 were key outliers; null result robust.
Quality of life	0.28 (0.04, 0.51)	0.02	18	0.01	83.9	The improvement following “digital health exercise” was comparable to “offline exercise”. Significant benefit for “Digital health exercise plus nutrition/health exercise plus education vs health education “.	Robust; no influential outliers.
Body mass index	−0.06 (−1.28, 1.17)	0.93	90	< 0.00001	95.6	Opposing effects: reduction (Digital health exercise/health exercise plus education vs. maintain original lifestyle/health education) vs. increase (Digital nutrition/health exercise plus nutrition vs. health education).	Robust; no influential outliers.

The meta-analysis demonstrated a statistically significant, moderate improvement in handgrip strength favoring digital interventions (MD = 1.07, 95% CI [0.30, 1.84], *p* = 0.007). Despite considerable heterogeneity (I² = 95%), subgroup analysis explained a substantial portion of this variance (*p* = 0.0004, I² = 83.3%), revealing a large, significant benefit specifically for comprehensive “digital exercise plus nutrition” interventions (MD = 2.21). Sensitivity analysis identified studies Y02(a) and Y02(b) as key sources of heterogeneity, though the overall positive conclusion remained robust.

2. Appendicular skeletal muscle index

For ASMI, the overall meta-analysis showed no statistically significant difference between digital interventions and control conditions (MD = 0.16, 95% CI [−0.03, 0.36], *p* = 0.10) with considerable heterogeneity observed (I² = 82%). Subgroup analysis was highly significant (*p* < 0.00001, I² = 94.1%), clarifying that digital exercise improved ASMI compared to “maintain original lifestyle” but was comparable to “offline exercise”. The removal of study Y05 in sensitivity analysis nullified the overall effect (MD = 0.11, *p* = 0.24) and reduced heterogeneity, identifying it as a major influential study.

3. Total skeletal muscle mass

Digital interventions significantly increased TSMM (MD = 0.90, 95% CI [0.31, 1.49], *p* = 0.003) with moderate heterogeneity (I² = 54%). Subgroup differences were significant (*p* = 0.004, I² = 87.9%), confirming that digital strategies were substantially more effective than maintain original lifestyle or health education. The finding was robust to sensitivity analysis; removing study Y04b attenuated the effect (MD = 0.73) but it remained significant, with a reduction in overall heterogeneity (I² = 44%).

4. Timed up-and-go test

No significant overall effect on TUGT performance was found (SMD = −0.02, 95% CI [−0.40, 0.37], *p* = 0.94). The analysis was plagued by substantial heterogeneity (I² = 80%), which was not explained by subgroup differences (*p* = 0.98). Sensitivity analysis identified Y02(b) as a critical outlier; its removal substantially reduced heterogeneity (I² = 52%) and shifted the overall estimate, but the conclusion of no significant effect remained robust.

5. Sit-to-stand test

A significant, small-to-moderate improvement in STS performance was found (MD = −0.52, 95% CI [−1.04, 0.00], *p* = 0.05), with low heterogeneity (I² = 43%). Subgroup analysis was highly informative (*p* = 0.007, I² = 86.2%), showing a large benefit for “digital exercise/health exercise plus nutrition” when compared with “maintain original lifestyle or health education”. The result was strengthened in sensitivity analysis; after removing Y04b, the effect became more pronounced and highly significant (MD = −0.71, *p* = 0.003), and heterogeneity dropped (I² = 23%).

6. Gait speed

The overall analysis showed a negligible, non-significant effect on gait speed (SMD = 0.06, 95% CI [−0.44, 0.56], *p* = 0.82). Extreme heterogeneity was present (I² = 84%) and not explained by subgroup differences (*p* = 0.30). A stepwise sensitivity analysis revealed that studies Y08 and Y15 were the primary drivers of this heterogeneity. After their removal, heterogeneity was fully resolved (I² = 0%), affirming a robust and consistent null effect (SMD = 0.04, *p* = 0.72).

7. Quality of life

A modest but statistically significant improvement in QoL was observed (SMD = 0.28, 95% CI [0.04, 0.51], *p* = 0.02), with low heterogeneity (I² = 18%). Subgroup analysis revealed that this benefit was driven by comprehensive digital interventions (*p* = 0.01, I² = 83.9%), as digital exercise alone showed no advantage over offline exercise. The finding was robust, with no single study unduly influencing the results.

8. Body mass index

The overall effect on BMI was negligible and non-significant (MD = −0.06, 95% CI [−1.28, 1.17], *p* = 0.93), with extreme heterogeneity (I² = 90%). However, subgroup analysis uncovered critically opposing effects (*p* < 0.00001, I² = 95.6%): digital health exercise/health exercise plus education interventions reduced BMI, while digital nutrition/health exercise plus nutrition interventions increased it. This subgroup effect was robust, with no influential outliers detected in sensitivity analysis.

9. Meta-analysis summary

The meta-analyses indicated a distinct pattern of efficacy for digital interventions across the outcome measures. Significant benefits were observed for handgrip strength and total skeletal muscle mass, alongside a modest improvement in quality of life, with subgroup analyses consistently favouring comprehensive interventions. The efficacy of digital interventions on the appendicular skeletal muscle mass Index and the sit-to-stand test was contingent upon the specific comparator. For appendicular skeletal muscle mass index, digital health exercise was superior to maintaining an original lifestyle but comparable to offline exercise. For the sit-to-stand test, significant benefits were observed for digital health exercise compared to maintaining an original lifestyle, as well as for digital health exercise plus nutrition compared to health education. In contrast, no significant overall effects were found for gait speed or the timed up-and-go test. A unique divergent effect was identified for body mass index, where subgroup analysis revealed that exercise-focused interventions reduced body mass index, while nutrition-focused interventions increased it.

### Publication bias

**Fig. 5:**
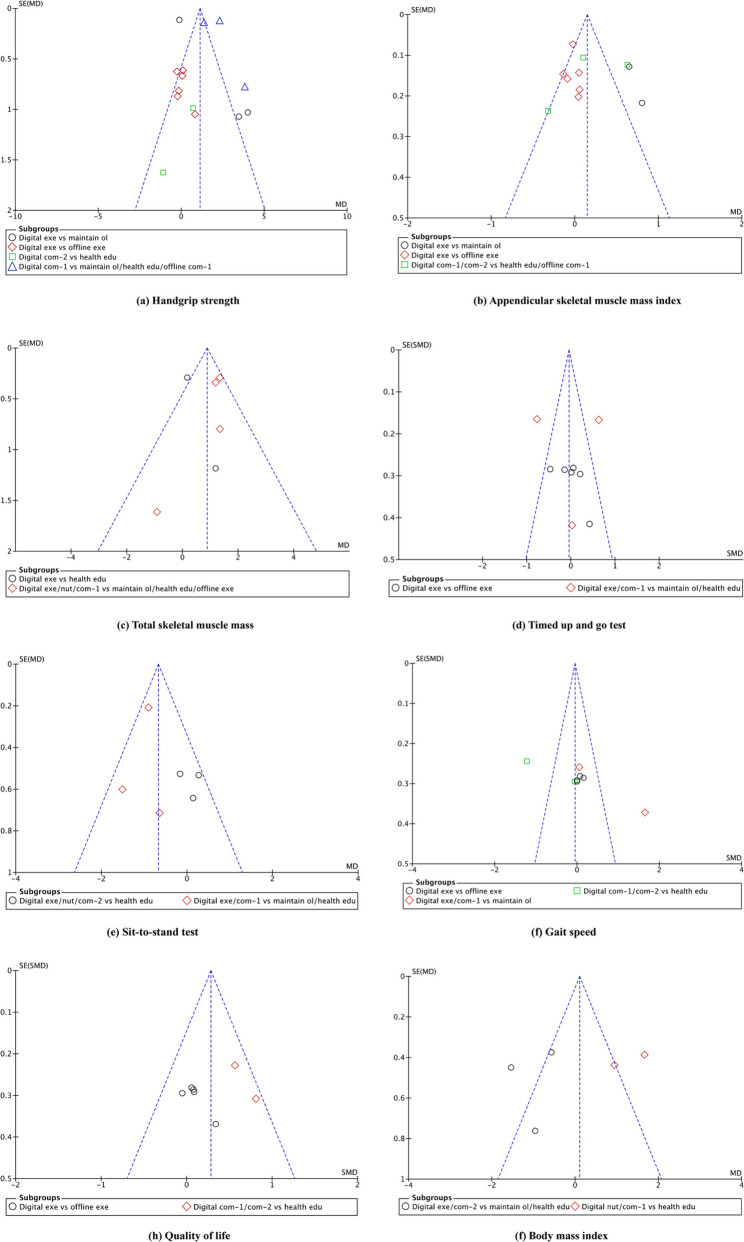
Funnel plots for the assessment of publication bias of all eight outcomes for sarcopenia. Note: The x-axis represents the mean difference or the standardised mean difference, and the y-axis represents the standard error. Digital exe — Digital health exercise; Digital nut — Digital nutrition; Digital com-1 — Digital health exercise + nutrition; Digital com-2 — Digital health exercise + education; Maintain ol — Maintain original lifestyle; Health edu — Health education; Offline exe — Offline exercise; Offline com-1 — Offline exercise + nutrition

The Funnel plots for the eight primary outcomes were visually inspected to assess the potential for publication bias (Fig. 5). The plots for majority of outcomes, including total skeletal muscle mass, timed up-and-go test, sit-to-stand test, gait speed, quality of life, and body mass index, demonstrated a roughly symmetrical distribution of effect estimates around the pooled mean. This symmetry suggests a low risk of publication bias for these outcomes.

Notable asymmetry was observed in the funnel plots for two outcomes: handgrip strength and appendicular skeletal muscle mass index. Visual inspection of the funnel plot for handgrip strength suggested asymmetry, characterized by a widely scattered distribution of effect sizes, consistent with the high heterogeneity in the meta-analysis. Studies from subgroups such as ‘Digital exercise vs maintain original lifestyle’ and ‘Digital exercise vs offline exercise’ showed a more concentrated spread, while others were represented by fewer data points. Despite the visual asymmetry, Egger’s regression test indicated no significant small-study effects (bias coefficient = 0.06, SE = 1.103, *P* = 0.959). For ASMI, the funnel plot appeared asymmetric, with an uneven distribution of studies, particularly in the lower half, across subgroups such as ‘digital exercise vs offline exercise’. Nonetheless, Egger’s regression test indicated no significant result (bias coefficient = −0.48, SE = 2.381, *P* = 0.841), which suggests no strong evidence that publication bias substantially influenced the overall findings of this meta-analysis.

#### Certainty of the evidence

The overall certainty of evidence across studies of the outcomes is presented in Supplementary Material 4. The overall body of evidence regarding the effectiveness of digital health interventions compared to traditional interventions for older adults with or at risk of sarcopenia is characterized by very low to low certainty. This assessment, based on the GRADE methodology, is consistent across the critical outcomes of handgrip strength, muscle mass, and physical performance.

For handgrip strength, the evidence is inconclusive. Most comparisons, including digital exercise versus offline exercise and digital communication/education versus health education, were supported by evidence of very low certainty. Key limitations included serious risk of bias, extreme inconsistency (I² up to 94%), and serious indirectness due to poorly defined sarcopenic populations. Although some analyses suggested a beneficial effect, the very wide confidence intervals that crossed the line of no effect render these results inconclusive for clinical decision-making.

For muscle mass, the findings are similarly uncertain. While one comparison (digital health exercise vs. maintain original lifestyle) showed a high-certainty benefit for ASMI, most comparisons for ASMI and total skeletal muscle mass were graded as very low certainty. This was primarily due to a very serious risk of bias, serious imprecision, and concerns regarding the indirectness of the study populations. Consequently, no reliable conclusions can be drawn regarding the superiority of either digital or traditional interventions for improving muscle mass.

For physical performance, measured by tests such as the timed up-and-go, sit-to-stand, and gait speed, the certainty of evidence was almost uniformly very low. The results were compromised by a very serious risk of bias, profound and unexplained statistical heterogeneity (I² up to 94%), and serious imprecision. The confidence intervals for nearly all comparisons were wide enough to include both clinically meaningful benefit and harm, indicating that the true effect of digital interventions remains unknown. The sole exception was for the sit-to-stand test in one comparison (digital health exercise/health exercise plus nutrition vs. maintain original lifestyle/health education), which demonstrated a moderate-certainty benefit, suggesting that digital interventions may be effective for this specific measure of lower limb strength.

In contrast, for secondary outcomes such as quality of life, the evidence was more robust for one specific comparison (digital health exercise plus nutrition/health exercise plus education vs. health education), which was graded as high certainty. However, for other comparisons and for body mass index, the certainty was low or very low, limiting broader conclusions.

In summary, the current evidence is insufficient to confidently determine whether digital interventions are superior, inferior, or equivalent to traditional interventions for improving most sarcopenia-related outcomes in older adults. The pervasive methodological limitations of the existing studies, particularly the high risk of bias and inconsistent results, substantially undermine the confidence in the pooled effect estimates.

## Discussion

This systematic review has several strengths that enhance the robustness and clinical relevance of its findings. First, a comprehensive search across 11 English and Chinese databases, without language or date restrictions, minimised selection bias and ensured broad evidence coverage [74]. Second, protocol pre-registration [90] and adherence to PRISMA guidelines [74] strengthened transparency and methodological rigor. Third, beyond a simple “digital versus non-digital” comparison, we conducted detailed subgroup analyses examining intervention type (e.g., exercise alone vs. combined exercise and nutrition) and comparator conditions, explaining substantial heterogeneity and yielding clinically actionable insights. Finally, the combined use of Cochrane RoB 2 [50] and ROBINS-I [52], separate appraisal of objective and subjective outcomes, and GRADE assessment [55] provided a rigorous evaluation of evidence quality and clarified the certainty of conclusions.

This review synthesized evidence from 16 studies published between 2017 and 2025, investigating the effectiveness of digital health exercise interventions on sarcopenia-related outcomes in older adults. The burgeoning number of publications in 2024 and 2025 indicates a rapidly evolving interest in this field, likely accelerated by technological advancements and a post-pandemic shift towards telehealth [75]. However, the heavy concentration of studies in China (68.75%), while demonstrating regional leadership, may limit the generalizability of our findings to other healthcare systems and cultural contexts with differing levels of digital infrastructure and exercise habits. Furthermore, the substantial variation in the diagnostic criteria for sarcopenia, with AWGS2 [7] predominating but not universally applied, impedes valid cross-study comparisons and fundamentally challenges the consistency of the population we are attempting to synthesize, potentially contributing to the observed heterogeneity.

This heterogeneity is further compounded by the diversity of the digital interventions themselves. The field encompasses a wide spectrum of tools, from video conferencing to AI-driven systems. While most interventions were methodologically robust in design, being largely guideline-based (75.00%), multicomponent (87.50%), and progressively intense, key limitations constrain the conclusions. The predominantly short-term duration (56.25% lasting 12 weeks) and notable scarcity of long-term follow-up (18.75%) fundamentally limit insights into the sustainability of any benefits [76]. Furthermore, the range of implementation models, from high-supervision to self-administered, suggests that “digital health” is not a monolithic intervention but a highly variable one, whose effectiveness is likely contingent on individual factors like technological literacy and functional status. Collectively, these factors constitute a dynamic but heterogeneous evidence base, which fundamentally underpins the “promising yet precarious” tension that is central to our findings.

A complete appreciation of the current evidence requires an understanding of the interplay between pooled effects and methodological limitations, which creates a situation of both promise and peril. Our meta-analysis demonstrated statistically significant benefits for several key outcomes, including handgrip strength, total skeletal muscle mass, and quality of life. This surface-level optimism, however, is immediately undercut by the risk of bias assessment. The fact that most included studies were rated as having “some concerns” or a “high risk” of bias overall fundamentally undermines the robustness of these findings. For instance, while objective outcomes like handgrip strength benefit from low risk of measurement bias, pervasive concerns in randomization processes and high rates of missing data suggest that the observed effects might be inflated [77]. The significant improvement in quality of life, a patient-reported outcome, is particularly vulnerable to bias due to the lack of blinding in over half of the studies [78]. Therefore, the positive pooled estimates must be interpreted as emerging from a methodologically fragile evidence base.

Given these methodological concerns, it is unsurprising that our findings are characterized by substantial heterogeneity, which itself constitutes a lack of a unified story and highlights the critical guidance offered by our subgroup analyses. This substantial, and often extreme, statistical heterogeneity (e.g., I² > 80% for handgrip strength and gait speed) is not merely a statistical nuisance but a core finding: the studies do not tell a single, consistent story. Fortunately, our subgroup analyses provided crucial clarity amidst this inconsistency. They successfully explained a substantial portion of heterogeneity for several outcomes. The most striking example was for BMI, where subgroup analysis uncovered diametrically opposing effects. The opposite effects of “exercise reduces BMI while nutrition increases BMI” identified in the BMI analysis reflect the complexity of body composition changes - exercise leads to fat loss while possibly causing a slight loss of muscle mass, while nutrition promotes muscle gain while potentially increasing fat [79]. This opposing effect is crucial when formulating combined interventions.

The superior efficacy of combined digital exercise and nutrition interventions observed in our meta-analysis, particularly for handgrip strength and quality of life, likely reflects synergistic physiological and behavioural mechanisms. Physiologically, resistance training activates mTOR-mediated muscle protein synthesis (MPS), while adequate protein intake, especially leucine-rich essential amino acids, provides the substrate necessary to sustain this anabolic response [80]. Concurrent delivery may therefore produce additive or multiplicative effects on net muscle protein accretion, explaining greater improvements in strength and mass compared with exercise alone [81]. Behaviourally, integrated digital platforms may enhance adherence through reminders, self-monitoring, and feedback, facilitating effective implementation of nutritional support [82]. Moreover, combining exercise and nutrition may better address energy balance: exercise alone may induce catabolic risk under insufficient intake, whereas nutrition without activity may increase fat mass [79, 83]. Their integrated digital delivery may optimise body composition, consistent with our BMI subgroup findings showing divergent effects between exercise-only and nutrition-inclusive interventions. Collectively, these mechanisms support the design of multicomponent digital programmes targeting both anabolic stimulation and substrate availability rather than exercise in isolation.

These analyses move beyond a simplistic “digital versus traditional” comparison toward a more clinically meaningful question: which digital strategy, for whom, and relative to what comparator. Nevertheless, substantial heterogeneity across several outcomes necessitates cautious interpretation of pooled estimates, which should be viewed as indicative rather than definitive, with greater emphasis placed on more consistent subgroup findings. Importantly, subgroup results remain hypothesis-generating, particularly where sample sizes were small, and some comparisons were based on few studies, increasing susceptibility to chance findings or residual confounding. Future trials should be prospectively designed to test these hypotheses with adequate power and prespecified stratification variables.

All the concerns like risk of bias, inconsistency, and imprecision, culminate in the GRADE assessment, which serves as the final arbiter for transitioning from “results we see” to “conclusions we believe.” The judgment of “very low” to “low” certainty for most outcomes and comparisons is the most definitive finding of our review. It means that our confidence in the effect estimates is limited, and the true effect may be substantially different from what we observed. For example, the promising effect on handgrip strength was downgraded due to serious risk of bias and inconsistency. The single exception, the high-certainty evidence for comprehensive digital interventions improving quality of life over health education alone, stands out as the most reliable and actionable finding, pointing towards the superior value of multi-component digital care [84]. Therefore, even statistically significant findings based on low- or very low-certainty evidence should be interpreted as tentative rather than confirmatory, and caution is warranted when translating these results into clinical recommendations.

Further compounding the uncertainty is the assessment of publication bias and the robustness of our findings. While Egger’s test did not reach statistical significance for most outcomes, visual asymmetry in funnel plots for handgrip strength and ASMI introduces an additional layer of caution; we cannot rule out the possibility that the overall effect estimates are somewhat optimistic due to unpublished null findings [85]. Furthermore, sensitivity analyses confirmed that the results for certain outcomes (e.g., TUGT, gait speed) were driven by influential studies, indicating a lack of robustness and reinforcing the precarious nature of the evidence base.

This review has several limitations, largely reflecting constraints of the primary studies. Overall methodological quality was suboptimal, with most outcomes rated as very low to low certainty, owing to concerns such as inadequate randomization, limited blinding (particularly for subjective outcomes), and attrition, which may affect pooled estimates. Substantial clinical and methodological heterogeneity, arising from varying diagnostic criteria, digital platforms and delivery modes, and exercise protocols, limits definitive, universal recommendations despite subgroup exploration. Generalizability is also constrained by the geographic concentration of studies, predominantly in East Asia (mainly China), with others from Europe and Korea; differences in healthcare systems, digital literacy, diet, and cultural attitudes toward technology may influence effectiveness [86]. Ethnic variation in body composition, reflected in population-specific criteria such as AWGS, further limits direct extrapolation across groups [87–89]. Moreover, the predominantly short intervention duration (median 12 weeks) leaves long-term sustainability uncertain. Future multinational trials with longer follow-up are warranted to clarify contextual influences and durability of effects.

## Conclusion

In conclusion, this review provides cautiously optimistic evidence supporting digital health interventions for sarcopenia, while underscoring critical evidence gaps. Three key findings emerge. First, integrated digital exercise-nutrition interventions show the most consistent improvements in muscle strength, mass, and quality of life, indicating synergistic benefits that merit prioritized investigation. Second, intervention effectiveness is comparator-dependent: digital exercise outperforms usual lifestyle maintenance but is generally comparable to offline exercise, suggesting its greatest value lies where supervised programmes are inaccessible rather than as a universal substitute. Third, while the promising effects support continued exploration, methodological strengthening through standardized interventions, longer-term follow-up, and precision medicine approaches is essential to establish definitive evidence. Clinically, digital multicomponent interventions may represent a practical alternative when traditional services are limited, particularly if tailored to individual patient needs.

## Supplementary Information

Supplementary Material 1-4

## Data Availability

The datasets analysed during the current study are available from the corresponding author on reasonable request.
