# Supplementary material for: Effectiveness of digital health exercise interventions on muscle function and physical performance in older adults with possible, confirmed, or severe sarcopenia: a systematic review and meta-analysis
Source: Eur Rev Aging Phys Act. 2026 Apr 10;23:15. doi: 10.1186/s11556-026-00409-x (PMC13154666; doi:10.1186/s11556-026-00409-x)
Supplement: Supplementary file 1 — Supplementary Material 1-4 [file 11556_2026_409_MOESM1_ESM.pdf]

SUPPLEMENT MATERIAL 1

RESULTS OF SEARCH STRATEGY

· WEB OF SCIENCE search strategy

NUM	SEARCH STRATEGY	RESULT
1	TS=(older adult* or older people or older person or elder* or geriatric* or senior* or senior citizen* or ag?ing or aged or age, eld* or oldest old or nonagenarian* or octogenarian* or centenarian* or pensioner*)	6469995
2	TS=(sarcopeni* or muscle mass or muscle strength or hand strength or grip strength or EWGSOP or AWGS)	252365
3	TS=(tele* or ehealth or e-health or digital health or mhealth or m-health or mobile health or remote health or remote care or remote teaching or remote training or remote exercise or remote nutrition or distance care or distance learning or distance education or web* or Internet or online or computer* or tablet* or smartphone* or app* or email* or video* or electronic or social media or Twitter or Facebook or Instagram or YouTube or WhatsApp or Microsoft Teams or Zoom or TikTok or WeChat or Weibo or QQ)	25041993
4	TS=(physical activit* or exercise* or sport* or training or coaching or tai chi or Tai Ji)	2882610
5	TS=(randomi?ed control* trial or RCT* or control* trial or clinical trial*)	1544151
6	#1 AND #2 AND #3 AND #4 AND #5	2989
7	TI=(qualitative stud* or cross-sectional stud* or cohort stud* or case-control stud* or review* or meta-analysis or protocol* or conference* or case report* or comment*)	2753622
8	(#6) NOT #7	1803

· MEDLINE search strategy (through WOS)

NUM	SEARCH STRATEGY	RESULT
1	older adult* or older people or older person or elder* or geriatric* or senior* or senior citizen* or ag?ing or aged or age, eld* or oldest old or nonagenarian* or octogenarian* or centenarian* or pensioner* (Topic)	9528115
2	sarcopeni* or muscle mass or muscle strength or hand strength or grip strength or EWGSOP or AWGS (Topic)	204214
3	tele* or ehealth or e-health or digital health or mhealth or m-health or mobile health or remote health or remote care or remote teaching or remote training or remote exercise or remote nutrition or distance care or distance learning or distance education or web* or Internet or online or computer* or tablet* or smartphone* or app* or email* or video* or electronic or social media or Twitter or Facebook or Instagram or YouTube or WhatsApp or Microsoft Teams or Zoom or TikTok or WeChat or Weibo or QQ (Topic)	10972212
4	physical activit* or exercise* or sport* or training or coaching or tai chi or Tai Ji (Topic)	1613428
5	randomi?ed control* trial or RCT* or control* trial or clinical trial* (Topic)	1448421
6	#1 AND #2 AND #3 AND #4 AND #5	2885
7	TI=(qualitative stud* or cross-sectional stud* or cohort stud* or case-control stud* or review* or meta-analysis or protocol* or conference* or case report* or comment*)	2037160
8	(#6) NOT #7	1911

· EMBASE search strategy

NUM	SEARCH STRATEGY	RESULT
1	'older adult*':ti,ab,kw OR 'older people':ti,ab,kw OR 'older person':ti,ab,kw OR elder*:ti,ab,kw OR geriatric*:ti,ab,kw OR senior*:ti,ab,kw OR 'senior citizen*':ti,ab,kw OR ag?ing:ti,ab,kw OR aged:ti,ab,kw OR 'age, eld*':ti,ab,kw OR 'oldest old':ti,ab,kw OR nonagenarian*:ti,ab,kw OR octogenarian*:ti,ab,kw OR centenarian*:ti,ab,kw OR pensioner*:ti,ab,kw	1956120
2	sarcopeni*:ti,ab,kw OR 'muscle mass':ti,ab,kw OR 'muscle strength':ti,ab,kw OR 'hand strength':ti,ab,kw OR 'grip strength':ti,ab,kw OR ewgsop:ti,ab,kw OR awgs:ti,ab,kw	139193
3	tele*:ti,ab,kw OR ehealth:ti,ab,kw OR 'e health':ti,ab,kw OR 'digital health':ti,ab,kw OR mhealth:ti,ab,kw OR 'm health':ti,ab,kw OR 'mobile health':ti,ab,kw OR 'remote health':ti,ab,kw OR 'remote care':ti,ab,kw OR 'remote teaching':ti,ab,kw OR 'remote training':ti,ab,kw OR 'remote exercise':ti,ab,kw OR 'remote nutrition':ti,ab,kw OR 'distance care':ti,ab,kw OR 'distance learning':ti,ab,kw OR 'distance education':ti,ab,kw OR web*:ti,ab,kw OR internet:ti,ab,kw OR online:ti,ab,kw OR computer*:ti,ab,kw OR tablet*:ti,ab,kw OR smartphone*:ti,ab,kw OR app*:ti,ab,kw OR email*:ti,ab,kw OR video*:ti,ab,kw OR electronic:ti,ab,kw OR 'social media':ti,ab,kw OR twitter:ti,ab,kw OR facebook:ti,ab,kw OR instagram:ti,ab,kw OR youtube:ti,ab,kw OR whatsapp:ti,ab,kw OR 'microsoft teams':ti,ab,kw OR zoom:ti,ab,kw OR tiktok:ti,ab,kw OR wechat:ti,ab,kw OR weibo:ti,ab,kw OR qq:ti,ab,kw	13174974

4	'physical activit*':ti,ab,kw OR exercise*:ti,ab,kw OR sport*:ti,ab,kw OR training:ti,ab,kw OR coaching:ti,ab,kw OR 'tai chi':ti,ab,kw OR 'tai ji':ti,ab,kw	1691768
5	'randomi?ed control* trial':ti,ab,kw OR rct*:ti,ab,kw OR 'control* trial':ti,ab,kw OR 'clinical trial*':ti,ab,kw	1316023
6	#1 AND #2 AND #3 AND #4 AND #5	1294
7	'qualitative stud*':ti OR 'cross-sectional stud*':ti OR 'cohort stud*':ti OR 'case-control stud*':ti OR review*:ti OR 'meta analysis':ti OR protocol*:ti OR conference*:ti OR 'case report*':ti OR comment*:ti	2273063
8	#6 NOT #7	971

· APA PSYCINFO search strategy

NUM	SEARCH STRATEGY	RESULT
1	Title: older adult* OR Title: older people OR Title: older person OR Title: elder* OR Title: geriatric* OR Title: senior* OR Title: senior citizen* OR Title: ag?ing OR Title: aged OR Title: age, eld* OR Title: oldest old OR Title: nonagenarian* OR Title: octogenarian* OR Title: centenarian* OR Title: pensioner* OR Keywords: older adult* OR Keywords: older people OR Keywords: older person OR Keywords: elder* OR Keywords: geriatric* OR Keywords: senior* OR Keywords: senior citizen* OR Keywords: ag?ing OR Keywords: aged OR Keywords: age, eld* OR Keywords: oldest old OR Keywords: nonagenarian* OR Keywords: octogenarian* OR Keywords: centenarian* OR Keywords: pensioner* OR Abstract: older adult* OR Abstract: older people OR Abstract: older person OR Abstract: elder* OR Abstract: geriatric* OR Abstract: senior* OR Abstract: senior citizen* OR Abstract: ag?ing OR Abstract: aged OR Abstract: age, eld* OR Abstract: oldest old OR Abstract: nonagenarian* OR Abstract: octogenarian* OR Abstract: centenarian* OR Abstract: pensioner*	1690931
2	Title: sarcopeni* OR Title: muscle mass OR Title: muscle strength OR Title: hand strength OR Title: grip strength OR Title: EWGSOP OR Title: AWGS OR Keywords: sarcopeni* OR Keywords: muscle mass OR Keywords: muscle strength OR Keywords: hand strength OR Keywords: grip strength OR Keywords: EWGSOP OR Keywords: AWGS OR Abstract: sarcopeni* OR Abstract: muscle mass OR Abstract: muscle strength OR Abstract: hand strength OR Abstract: grip strength OR Abstract: EWGSOP OR Abstract: AWGS	9891
3	Title: tele* OR Title: ehealth OR Title: e-health OR Title: digital health OR Title: mhealth OR Title: m-health OR Title: mobile health OR Title: remote health OR Title: remote care OR Title: remote teaching OR Title: remote training OR Title: remote exercise OR Title: remote nutrition OR Title: distance care OR Title: distance learning OR Title: distance education OR Title: web* OR Title: Internet OR Title: online OR Title: computer* OR Title: tablet* OR Title: smartphone* OR Title: app* OR Title: email* OR Title: video* OR Title: electronic OR Title: social media OR Title: Twitter OR Title: Facebook OR Title: Instagram OR Title: YouTube OR Title: WhatsApp OR Title: Microsoft Teams OR Title: Zoom OR Title: TikTok OR Title: WeChat OR Title: Weibo OR Title: QQ OR Keywords: tele* OR Keywords: ehealth OR Keywords: e-health OR Keywords: digital health OR Keywords: mhealth OR Keywords: m-health OR Keywords: mobile health OR Keywords: remote health OR Keywords: remote care OR Keywords: remote teaching OR Keywords: remote training OR Keywords: remote exercise OR Keywords: remote nutrition OR Keywords: distance care OR Keywords: distance learning OR Keywords: distance education OR Keywords: web* OR Keywords: Internet OR Keywords: online OR Keywords: computer* OR Keywords: tablet* OR Keywords: smartphone* OR Keywords: app* OR Keywords: email* OR Keywords: video* OR Keywords: electronic OR Keywords: social media OR Keywords: Twitter OR Keywords: Facebook OR Keywords: Instagram OR Keywords: YouTube OR Keywords: WhatsApp OR Keywords: Microsoft Teams OR Keywords: Zoom OR Keywords: TikTok OR Keywords: WeChat OR Keywords: Weibo OR Keywords: QQ OR Abstract: tele* OR Abstract: ehealth OR Abstract: e-health OR Abstract: digital health OR Abstract: mhealth OR Abstract: m-health OR Abstract: mobile health OR Abstract: remote health OR Abstract: remote care OR Abstract: remote teaching OR Abstract: remote training OR Abstract: remote exercise OR Abstract: remote nutrition OR Abstract: distance care OR Abstract: distance learning OR Abstract: distance education OR Abstract: web* OR Abstract: Internet OR Abstract: online OR Abstract: computer* OR Abstract: tablet* OR Abstract: smartphone* OR Abstract: app* OR Abstract: email* OR Abstract: video* OR Abstract: electronic OR Abstract: social media OR Abstract: Twitter OR Abstract: Facebook OR Abstract: Instagram OR Abstract: YouTube OR Abstract: WhatsApp OR Abstract: Microsoft Teams OR Abstract: Zoom OR Abstract: TikTok OR Abstract: WeChat OR Abstract: Weibo OR Abstract: QQ	2317010
4	Title: physical activit* OR Title: exercise* OR Title: sport* OR Title: training OR Title: coaching OR Title: tai chi OR Title: Tai Ji OR Keywords: physical activit* OR Keywords: exercise* OR Keywords: sport* OR Keywords: training OR Keywords: coaching OR Keywords: tai chi OR Keywords: Tai Ji OR Abstract:	578674

	physical activit* OR Abstract: exercise* OR Abstract: sport* OR Abstract: training OR Abstract: coaching OR Abstract: tai chi OR Abstract: Tai Ji	
5	Title: randomi?ed control* trial OR Title: RCT* OR Title: control* trial OR Title: clinical trial* OR Keywords: randomi?ed control* trial OR Keywords: RCT* OR Keywords: control* trial OR Keywords: clinical trial* OR Abstract: randomi?ed control* trial OR Abstract: RCT* OR Abstract: control* trial OR Abstract: clinical trial*	135946
6	1 and 2 and 3 and 4 and 5	105
7	Title: qualitative stud* OR Title: cross-sectional stud* OR Title: cohort stud* OR Title: case-control stud* OR Title: review* OR Title: meta-analysis OR Title: protocol* OR Title: conference* OR Title: case report* OR Title: comment*	349996
8	6 not 7	80

· CINAHL search strategy

NUM	SEARCH STRATEGY	RESULT
S1	((MH "Sarcopenia") OR sarcopenia OR sarcopenic) OR SU (muscle mass or muscle strength or hand strength or grip strength or EWGSOP or AWGS)	82287
S2	SU (older adult* or older people or older person or elder* or geriatric* or senior* or senior citizen* or ag?ing or aged or age, eld* or oldest old or nonagenarian* or octogenarian* or centenarian* or pensioner*)	2041802
S3	SU (tele* or ehealth or e-health or digital health or mhealth or m-health or mobile health or remote health or remote care or remote teaching or remote training or remote exercise or remote nutrition or distance care or distance learning or distance education or web* or Internet or online or computer* or tablet* or smartphone* or app* or email* or video* or electronic or social media or Twitter or Facebook or Instagram or YouTube or WhatsApp or Microsoft Teams or Zoom or TikTok or WeChat or Weibo or QQ)	1101606
S4	SU (physical activit* or exercise* or sport* or training or coaching or tai chi or Tai Ji)	595044
S5	SU (randomi?ed control* trial or RCT* or control* trial or clinical trial*)	667660
S6	S1 AND S2 AND S3 AND S4 AND S5	56
S7	TI (qualitative stud* or cross-sectional stud* or cohort stud* or case-control stud* or review* or meta-analysis or protocol* or conference* or case report* or comment*)	1630749
S8	S6 NOT S7	51

· SCOPUS search strategy

NUM	SEARCH STRATEGY	RESULT
1	TITLE-ABS-KEY ((older AND adult*) OR (older AND people) OR (older AND person) OR elder* OR geriatric* OR senior* OR (senior AND citizen*) OR ag?ing OR aged OR (age, AND eld*) OR (oldest AND old) OR nonagenarian* OR octogenarian* OR centenarian* OR pensioner*)	9225232
2	TITLE-ABS-KEY (sarcopeni* OR (muscle AND mass) OR (muscle AND strength) OR (hand AND strength) OR (grip AND strength) OR EWGSOP OR AWGS)	393771
3	TITLE-ABS-KEY (tele* OR ehealth OR e-health OR (digital AND health) OR mhealth OR m-health OR (mobile AND health) OR (remote AND health) OR (remote AND care) OR (remote AND teaching) OR (remote AND training) OR (remote AND exercise) OR (remote AND nutrition) OR (distance AND care) OR (distance AND learning) OR (distance AND education) OR web* OR Internet OR online OR computer* OR tablet* OR smartphone* OR app* OR email* OR video* OR electronic OR (social AND media) OR Twitter OR Facebook OR Instagram OR YouTube OR WhatsApp OR (Microsoft AND Teams) OR Zoom OR TikTok OR WeChat OR Weibo OR QQ)	37795681
4	TITLE-ABS-KEY ((physical AND activit*) OR exercise* OR sport* OR training OR coaching OR (tai AND chi) OR (Tai AND Ji))	3604886
5	TITLE-ABS-KEY ((randomi?ed AND control* AND trial) OR RCT* OR (control* AND trial) OR (clinical AND trial*))	2877236
6	#1 AND #2 AND #3 AND #4 AND #5	5779
7	TITLE ((qualitative AND stud*) OR (cross-sectional AND stud*) OR (cohort AND stud*) OR (case-control AND stud*) OR review* OR meta-analysis OR protocol* OR conference* OR (case AND report*) OR comment*)	3297500
8	#6 AND NOT #7	4244

· COCHRANE LIBRARY search strategy

NUM	SEARCH STRATEGY	RESULT
1	sarcopenia or sarcopenic or (muscle mass) or (muscle strength) or (hand strength) or (grip strength) or EWGSOP or AWGS in Title Abstract Keyword	50569
2	AND (older adult*) or (older people) or (older person) or elder* or geriatric* or senior* or (senior citizen*)	30531

	or ag?ing or aged or (age, eld*) or oldest old or nonagenarian* or octogenarian* or centenarian* or pensioner* in Title Abstract Keyword	
3	AND tele* or ehealth or e-health or (digital health) or mhealth or m-health or (mobile health) or (remote health) or (remote care) or (remote teaching) or (remote training) or (remote exercise) or (remote nutrition) or (distance care) or (distance learning) or (distance education) or web* or Internet or online or computer* or tablet* or smartphone* or app* or email* or video* or electronic or (social media) or Twitter or Facebook or Instagram or YouTube or WhatsApp or Microsoft Teams or Zoom or TikTok or WeChat or Weibo or QQ in Title Abstract Keyword	12431
4	AND (physical activit*) or exercise* or sport* or training or coaching or (tai chi) or (Tai Ji) in Title Abstract Keyword	8564
5	AND (randomi?ed control* trial) or RCT* or (control* trial) or (clinical trial*) in Title Abstract Keyword	5032
6	NOT (qualitative stud*) or (cross-sectional stud*) or (cohort stud*) or (case-control stud*) or review* or meta-analysis or protocol* or conference* or (case report*) or comment* in Record Title	4558

· Four Chinese databases search results

NUM	Chinese databases	RESULT
1	CNKI (Before-2024.07)	171
2	WANFANG (Before-2024.07)	215
3	VIP (Before-2024.07)	170
4	CBM (Before-2024.07)	151

· GOOGLE SCHOLAR search strategy

NUM	SEARCH STRATEGY	RESULT
1	(sarcopenia OR sarcopenic) AND ((older adult*) OR (older person) OR (older people) OR elder* OR geriatric* OR senior*) AND (tele* OR ehealth OR e-health OR (digital health) OR mhealth OR m-health OR (mobile health) OR web* OR Internet OR online OR computer* OR tablet* OR smartphone* OR app* OR email* OR video* OR electronic OR (social media) OR Twitter OR Facebook OR Instagram OR YouTube OR WhatsApp OR Microsoft Teams OR Zoom OR TikTok OR WeChat OR Weibo OR QQ) AND ((physical activit*) OR exercise* OR sport* OR training OR coaching OR (tai chi) OR (Tai Ji)) AND ((randomi?ed control* trial) OR RCT* OR (control* trial) or (clinical trial*))	856

Supplementary Material 2. Intervention characteristics of the included studies

Code	Group	n (complete/recruit)	Mean age	Sex (F, %)	Mode	Frequency	Contents
Y1	Exercise	11/13	82.2 ± 5.6	6(54.5)	A 15-in. all-in-one PC and video conferencing software (Skype™)	3 sessions/week on non-consecutive days (separated by at least 48 h)	Resistance training
	Control	12/13	81.5 ± 4.4	7(58.3)	Offline	1 session/4-week	Health education (nutrition and exercise)
Y2	Exercise (a)	48/56/65	72.3 ± 5.8	43 (66.2)	A tablet PC with developed app (exercise) + Video call with Skype or phone call (exercise and nutrition)	≥ 2 sessions/week	Functional training
	Exercise + Nutrition (b)	43/47/68	70.8 ± 6.8	49 (72.1)		1-2 sessions/week	Dietary protein counselling 1.2-1.5 g/kg BW/day
	Control	77/81/91	72.8 ± 6.5	66 (72.5)	Offline	None	Maintain original lifestyle
Y3	Exercise (a)	48/55/63	72.3 ± 5.5	42 (66.7)	This study was conducted as part of the same research project as the previous study[58], but represents a different phase of the project. The intervention content and procedures were identical.		
	Eexercise + Nutrition (b)	42/45/65	71.0 ± 6.6	47 (72.3)			
	Control	77/81/84	73.0 ± 6.5	59 (70.2)			
Y4	Exercise (a)	50/62	69.72 ± 3.60	40 (80.0)	A smartphone app	≥ 3 sessions/week	Resistance + aerobic training
	Nutrition (b)	50/58	68.18 ± 3.93	41 (82.0)		Daily adjustment	Diet protein intake 1.0~1.5 g/kg BW/day (high-quality protein better reach 50%)
	Exercise + Nutrition (c)	50/60	70.16 ± 4.32	42 (84.0)		The specific content was consistent with the above two intervention groups.	
	Control	51/54	69.88 ± 3.29	44 (86.3)	Offline	Once	Health education on sarcopenia
Y5	Exercise + Nutrition	72/72	72.14 ± 5.06	29(40.3)	A web-based platform with both desktop and mobile interfaces	Not mentioned (exercise) + Daily adjustment (nutrition)	Individualized nutrition and exercise (resistance and aerobic training) plans
	Control	72/72	72.49 ± 5.24	32(44.4)	offline	The same content as the above intervention group.	
Y6	Exercise	28/30	78.83 ± 7.71	20 (66.7)	Nintendo's Ring Fit Adventure (RFA) for the Nintendo Switch	2 sessions/week on non-consecutive days (separated by at least 48 h)	Exergame-based resistance + aerobic training + functional movements
	Control	27/30	78.73 ± 6.82	19 (63.3)	offline	None	Maintain original lifestyle
Y7	Exercise	65/?	Not mentioned	Not mentioned	A GYM Platform consists of a smartphone app, a wearable device, and a dedicated website.	4 sessions/week	Resistance training
	Control	64/?	Not mentioned	Not mentioned	Offline	None	Maintain original lifestyle
Y8	Exercise + Nutrition	40/40	Not mentioned	20(50.0)	WeChat	3 sessions/week (exercise) + Daily adjustment (nutrition)	Exercise: resistance + aerobic training Nutrition: customized recipe
	Control	40/40	Not mentioned	17(42.5)	Offline	3 sessions/week	Health education (face-to-face + brochure + phone call)
Y9	Exercise	23/25	72.26 ± 4.43	Not mentioned	Tencent conferencing software	3 sessions/week	Taichi exercise videos while a professional instructor

	(a)						guided the movements
	Exercise (b)	24/25	73.67 ± 4.77	Not mentioned	MediaPipe (Google) + BlazePose + OpenCV		Taichi videos while the AI guided the movements
	Control	23/25	70.91 ± 3.94	Not mentioned	Offline		Face-to-face Taichi exercise
Y10	Exercise + Education	27/27	70.26 ± 4.72	24 (88.9)	A wearable activity tracker (Asus VivoWatch BP) + a smartphone app	Daily (exercise) 1 session/week (health education)	Exercise: walking Health education: group-based nutritional education about healthy eating
	Control	31/31	74.26 ± 6.30	25 (80.7)	Offline	1 session/week	Group-based nutritional health education about healthy eating
Y11	Exercise + Education	22/23	75.00 (70.00, 78.00)	20 (87.0)	An online platform + wearable monitoring devices	5 sessions/week	Exercise: resistance + aerobic + balance training Health education: sarcopenia and diabetes prevention
	Control	22/23	74.00 (72.00, 76.50)	19 (82.6)	Offline	1 session/week	Health education on sarcopenia and diabetes
Y12	Exercise	24/29	70.47 ± 6.05	18 (75.0)	An online platform + a smartphone app	3 sessions/week	Resistance training
	Control	27/29	69.81 ± 5.76	20 (74.1)	Offline	Face-to-face exercise: the specific content was consistent with the above intervention group.	
Y13	Exercise	15/15	76.24 ± 8.7	15(100.0)	Mixed Reality–Based Physical Therapy (Mr.PT) platform	3 sessions/week	A variety of functional and cognitive task exercises
	Control	15/15	74.88 ± 9.1	15(100.0)	Offline	3 sessions/week	Face-to-face exercise: gait + mobility + resistance + balance training
Y14	Exercise (a)	25/31	72.56 ± 7.76	12(48.0)	Tencent conferencing software		Yi Jin Jing exercise videos while a professional instructor guided the movements
	Exercise (b)	27/31	71.57 ± 7.24	13(48.1)	MediaPipe (Google) + BlazePose + OpenCV	3 sessions/week	Yi Jin Jing exercise videos while the AI guided the movements
	Control	24/31	70.77 ± 8.27	10(41.7)	Offline		Face-to-face Yi Jin Jing exercise
Y15	Exercise	40/40	Not mentioned	20(50.0)	Wearable devices (Garmin Vivosmart HR and Apple Watch)	5 sessions/week	Aerobic training: walking
	Control	40/40	Not mentioned	20(50.0)	Offline	None	Maintain original lifestyle
Y16	Exercise	13/13/15	75.80 ± 6.99	8 (46.7)	Nintendo Switch	2 sessions/week	Exergaming with resistance training
	Control	11/11/15	76.13 ± 8.16	7 (53.3)	Offline	2 sessions/week	Face-to-face resistance training

Note: "?" means data not reported. The letters a, b, and c respectively represent the different digital intervention groups in the included studies.

SUPPLEMENT MATERIAL 3

RESULT DATA SUPPLEMENT

Handgrip strength

· *Effect sizes*

The pooled effect estimates across 14 pairs of intervention indicated that digital interventions were associated with a statistically significant, moderate improvement in the outcome of interest compared to non-digital controls (Mean Difference = 1.07, 95% CI [0.30, 1.84], $p = 0.007$), as shown in Figure 4 (a).

However, the magnitude and direction of effects varied considerably across the predefined subgroups. In the "1.1.1 Digital exercise vs maintain original lifestyle" subgroup, the point estimate favored digital exercise interventions (MD = 2.34), but the result was not statistically significant (95% CI [-0.78, 5.46], $p = 0.14$). The "1.1.2 Digital exercise vs offline exercise" subgroup showed a minimal and non-significant effect, suggesting comparable outcomes between digital and offline exercise (MD = 0.00, 95% CI [-0.58, 0.59]). The "1.1.3 Digital health exercise plus education vs health education" subgroup also demonstrated a small, non-significant effect (MD = 0.24, 95% CI [-1.42, 1.90]). In contrast, the "1.1.4 Digital health exercise plus nutrition vs maintain original lifestyle/health education/offline health exercise plus nutrition" subgroup showed a large and statistically significant positive effect in favor of the comprehensive digital interventions (MD = 2.21, 95% CI [1.33, 3.09], $p < 0.00001$).

· *Heterogeneity analysis*

Considerable and statistically significant heterogeneity was observed across the included studies. The overall analysis yielded an I^2 statistic of 95% ($Tau^2 = 1.58$, Chi^2 $p < 0.00001$), indicating that approximately 95% of the variability in effect estimates is due to genuine differences between studies rather than sampling error. Significant heterogeneity was also present within all subgroups, with I^2 values ranging from 0% to 94%. This high level of heterogeneity necessitates a cautious interpretation of the pooled overall estimate and underscores the importance of the subgroup analysis and sensitivity analysis.

· *Subgroup analysis*

Subgroup analysis was conducted to explore potential sources of the high

heterogeneity. The test for subgroup differences was statistically significant (Chi^2 = 18.02, df = 3, p = 0.0004, I^2 = 83.3%), indicating that the type of intervention and control group explains a substantial portion (83.3%) of the observed variance. The analysis reveals that the effect of digital interventions is highly dependent on their specific design and the nature of the control comparator.

· Sensitivity analysis

A leave-one-out sensitivity analysis was performed to assess the robustness of the pooled results and to identify potential outliers. The analysis revealed that the overall conclusion—a statistically significant benefit of digital interventions—was robust (all pooled p-values ≤ 0.008). However, the magnitude of heterogeneity and specific subgroup findings were sensitive to the removal of particular studies, as shown in Supplemental Material 2 (a).

Removal of Study Y02(a): Excluding this study from the "1.1.1 Digital exercise vs maintain original lifestyle" subgroup resulted in a substantial change. The subgroup's effect became statistically significant (MD = 3.77, 95% CI [2.31, 5.22], $p < 0.00001$), and the heterogeneity within this subgroup was completely resolved (I^2 = 0%). Consequently, the overall heterogeneity across the meta-analysis decreased substantially from I^2 = 95% to I^2 = 85%.

Removal of Studies Y02(a) and Y02(b): Further excluding study Y02(b) from the "1.1.4 Digital health exercise plus nutrition vs maintain original lifestyle/health education/offline health exercise plus nutrition" subgroup led to an additional reduction in overall heterogeneity (I^2 = 75%). The effect estimates for this subgroup remained significant but was attenuated and less precise (MD = 2.48, 95% CI [0.09, 4.87], p = 0.04), with high residual heterogeneity (I^2 = 90%).

In summary, while the pooled positive effect of digital interventions was consistent, the studies from the Y02 trial (specifically Y02a and Y02b) were identified as influential outliers. Their inclusion was a major source of the high statistical heterogeneity observed in the primary analysis and significantly impacted the point estimates and confidence intervals of their respective subgroups.

(a) Handgrip strength - sensitivity analysis

Study or Subgroup	Digital Mean	Digital SD	Digital Total	Non-digital Mean	Non-digital SD	Non-digital Total	Weight	Mean Difference IV, Random, 95% CI
**1.1.1 Digital exe vs maintain ol**								
Y02 (a)	−0.5	0.7	65	−0.4	0.76	91	0.0%	−0.10 [−0.33, 0.13]
Y06	6.29	4.57	30	2.8	3.69	30	5.5%	3.49 [1.39, 5.59]
Y15	3.72	4.78	40	−0.3	4.42	40	5.8%	4.02 [2.00, 6.04]
**Subtotal (95% CI)**			**70**			**70**	**11.3%**	**3.77 [2.31, 5.22]**
Heterogeneity: $Tau^2 = 0.00$; $Chi^2 = 0.13$, df = 1 (P = 0.72); $I^2 = 0\%$								
Test for overall effect: Z = 5.07 (P < 0.00001)								
**1.1.2 Digital exe vs offline exe**								
Y09 (a)	−0.47	2.16	23	−0.2	2.09	23	8.9%	−0.27 [−1.50, 0.96]
Y09 (b)	−0.08	2.11	24	−0.2	2.09	23	9.0%	0.12 [−1.08, 1.32]
Y12	1.82	3.64	24	1	3.82	27	5.7%	0.82 [−1.23, 2.87]
Y14 (a)	0.62	3.45	25	0.85	2.58	24	6.9%	−0.23 [−1.93, 1.47]
Y14 (b)	0.94	2.14	27	0.85	2.58	24	8.5%	0.09 [−1.22, 1.40]
Y16	0.52	1.99	13	0.68	1.99	11	7.3%	−0.16 [−1.76, 1.44]
**Subtotal (95% CI)**			**136**			**132**	**46.3%**	**0.00 [−0.58, 0.59]**
Heterogeneity: $Tau^2 = 0.00$; $Chi^2 = 0.97$, df = 5 (P = 0.97); $I^2 = 0\%$								
Test for overall effect: Z = 0.01 (P = 0.99)								
**1.1.3 Digital com-2 vs health edu**								
Y10	−0.38	5.75	27	0.7	6.63	31	3.2%	−1.08 [−4.27, 2.11]
Y11	0.53	3.31	23	−0.2	3.4	23	6.1%	0.73 [−1.21, 2.67]
**Subtotal (95% CI)**			**50**			**54**	**9.2%**	**0.24 [−1.42, 1.90]**
Heterogeneity: $Tau^2 = 0.00$; $Chi^2 = 0.90$, df = 1 (P = 0.34); $I^2 = 0\%$								
Test for overall effect: Z = 0.28 (P = 0.78)								
**1.1.4 Digital com-1 vs maintain ol/health edu/offline com-1**								
Y02 (b)	1.9	0.76	68	−0.4	0.76	91	12.8%	2.30 [2.06, 2.54]
Y05	5.37	0.85	72	4	0.82	72	12.7%	1.37 [1.10, 1.64]
Y08	6.22	3.39	40	2.4	3.53	40	7.6%	3.82 [2.30, 5.34]
**Subtotal (95% CI)**			**180**			**203**	**33.1%**	**2.21 [1.33, 3.09]**
Heterogeneity: $Tau^2 = 0.48$; $Chi^2 = 31.37$, df = 2 (P < 0.00001); $I^2 = 94\%$								
Test for overall effect: Z = 4.91 (P < 0.00001)								
**Total (95% CI)**			**436**			**459**	**100.0%**	**1.21 [0.56, 1.86]**
Heterogeneity: $Tau^2 = 0.85$; $Chi^2 = 81.63$, df = 12 (P < 0.00001); $I^2 = 85\%$								
Test for overall effect: Z = 3.63 (P = 0.0003)								
Test for subgroup differences: $Chi^2 = 32.93$, df = 3 (P < 0.00001), $I^2 = 90.9\%$								

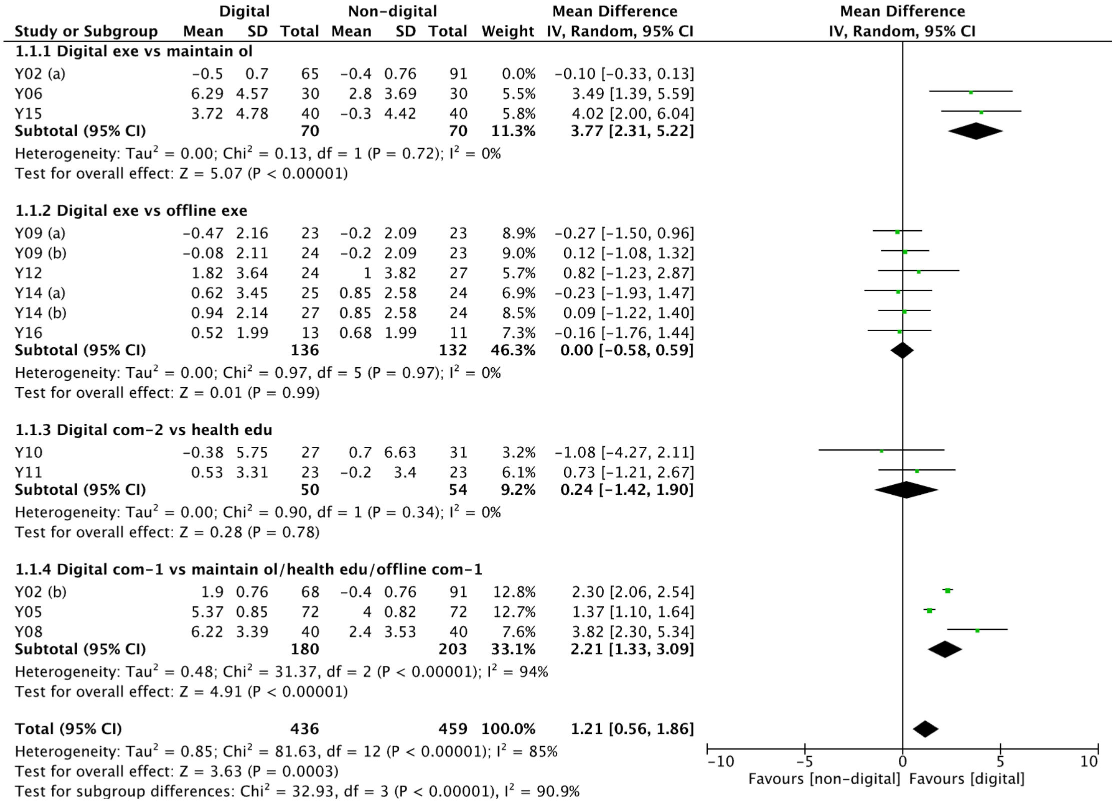

Study or Subgroup	Digital Mean	Digital SD	Digital Total	Non-digital Mean	Non-digital SD	Non-digital Total	Weight	Mean Difference IV, Random, 95% CI
**1.1.1 Digital exe vs maintain ol**								
Y02 (a)	−0.5	0.7	65	−0.4	0.76	91	0.0%	−0.10 [−0.33, 0.13]
Y06	6.29	4.57	30	2.8	3.69	30	6.7%	3.49 [1.39, 5.59]
Y15	3.72	4.78	40	−0.3	4.42	40	7.0%	4.02 [2.00, 6.04]
**Subtotal (95% CI)**			**70**			**70**	**13.7%**	**3.77 [2.31, 5.22]**
Heterogeneity: $Tau^2 = 0.00$; $Chi^2 = 0.13$, df = 1 (P = 0.72); $I^2 = 0\%$								
Test for overall effect: Z = 5.07 (P < 0.00001)								
**1.1.2 Digital exe vs offline exe**								
Y09 (a)	−0.47	2.16	23	−0.2	2.09	23	9.9%	−0.27 [−1.50, 0.96]
Y09 (b)	−0.08	2.11	24	−0.2	2.09	23	10.1%	0.12 [−1.08, 1.32]
Y12	1.82	3.64	24	1	3.82	27	6.9%	0.82 [−1.23, 2.87]
Y14 (a)	0.62	3.45	25	0.85	2.58	24	8.1%	−0.23 [−1.93, 1.47]
Y14 (b)	0.94	2.14	27	0.85	2.58	24	9.6%	0.09 [−1.22, 1.40]
Y16	0.52	1.99	13	0.68	1.99	11	8.5%	−0.16 [−1.76, 1.44]
**Subtotal (95% CI)**			**136**			**132**	**53.1%**	**0.00 [−0.58, 0.59]**
Heterogeneity: $Tau^2 = 0.00$; $Chi^2 = 0.97$, df = 5 (P = 0.97); $I^2 = 0\%$								
Test for overall effect: Z = 0.01 (P = 0.99)								
**1.1.3 Digital com-2 vs health edu**								
Y10	−0.38	5.75	27	0.7	6.63	31	4.1%	−1.08 [−4.27, 2.11]
Y11	0.53	3.31	23	−0.2	3.4	23	7.3%	0.73 [−1.21, 2.67]
**Subtotal (95% CI)**			**50**			**54**	**11.4%**	**0.24 [−1.42, 1.90]**
Heterogeneity: $Tau^2 = 0.00$; $Chi^2 = 0.90$, df = 1 (P = 0.34); $I^2 = 0\%$								
Test for overall effect: Z = 0.28 (P = 0.78)								
**1.1.4 Digital com-1 vs maintain ol/health edu/offline com-1**								
Y02 (b)	1.9	0.76	68	−0.4	0.76	91	0.0%	2.30 [2.06, 2.54]
Y05	5.37	0.85	72	4	0.82	72	13.0%	1.37 [1.10, 1.64]
Y08	6.22	3.39	40	2.4	3.53	40	8.8%	3.82 [2.30, 5.34]
**Subtotal (95% CI)**			**112**			**112**	**21.8%**	**2.48 [0.09, 4.87]**
Heterogeneity: $Tau^2 = 2.69$; $Chi^2 = 9.71$, df = 1 (P = 0.002); $I^2 = 90\%$								
Test for overall effect: Z = 2.03 (P = 0.04)								
**Total (95% CI)**			**368**			**368**	**100.0%**	**1.06 [0.28, 1.83]**
Heterogeneity: $Tau^2 = 1.18$; $Chi^2 = 44.07$, df = 11 (P < 0.00001); $I^2 = 75\%$								
Test for overall effect: Z = 2.67 (P = 0.008)								
Test for subgroup differences: $Chi^2 = 24.71$, df = 3 (P < 0.0001), $I^2 = 87.9\%$								

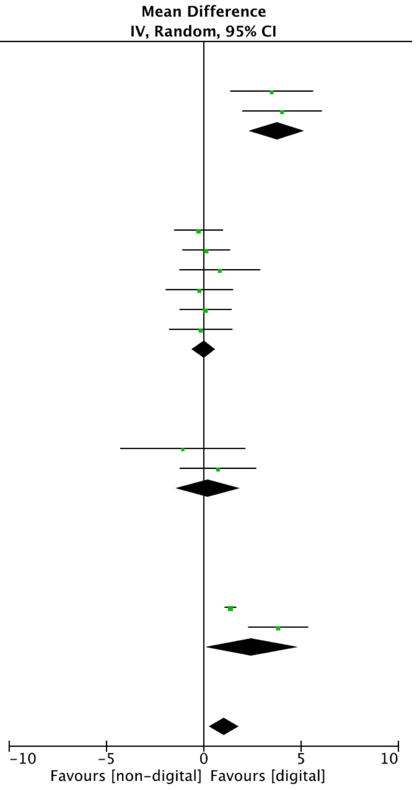

Appendicular skeletal muscle mass index

· *Effect sizes*

The overall pooled analysis across 11 pairs of intervention indicated a small, non-significant positive trend favoring digital interventions over non-digital controls for improving ASMI (Mean Difference [MD] = 0.16, 95% CI [-0.03, 0.36], $p = 0.10$). The clinical interpretation of this effect is uncertain due to its lack of statistical significance and the presence of substantial heterogeneity, as shown in Figure 4 (b).

The magnitude and direction of effects varied considerably across the predefined subgroups. In the "1.2.1 Digital exercise vs maintain original lifestyle" subgroup, the effect was statistically significant and substantial, favoring digital exercise interventions (MD = 0.69, 95% CI [0.47, 0.91], $p < 0.00001$). The "1.2.2 Digital exercise vs offline exercise" subgroup showed a minimal and non-significant effect, suggesting comparable outcomes between digital and offline exercise (MD = -0.02, 95% CI [-0.12, 0.08], $p = 0.65$). The "1.2.3 Digital health exercise plus nutrition/health exercise plus education vs health education/offline health exercise plus nutrition" subgroup demonstrated a non-significant positive effect (MD = 0.17, 95% CI [-0.31, 0.65], $p = 0.49$).

· *Heterogeneity analysis*

Considerable and statistically significant heterogeneity was observed across the included studies. The overall analysis yielded an I^2 statistic of 82% ($Tau^2 = 0.09$, Chi^2 $p < 0.00001$), indicating that a majority of the variability in effect estimates is due to genuine differences between studies. Heterogeneity was low ($I^2 = 0\%$) in the first two subgroups but high in the digital comprehensive intervention subgroup ($I^2 = 88\%$).

· *Subgroup analysis*

Subgroup analysis was conducted to explore potential sources of the high heterogeneity. The test for subgroup differences was statistically significant ($Chi^2 = 33.99$, $df = 2$, $p < 0.00001$, $I^2 = 94.1\%$), indicating that the type of intervention and control group explains a substantial portion (94.1%) of the observed variance. The analysis reveals that the effect on ASMI is highly dependent on the comparator, with a clear, significant benefit for digital exercise only when compared to maintaining original lifestyle, but not when compared to active, offline exercise interventions.

· Sensitivity analysis

A leave-one-out sensitivity analysis was performed to assess the robustness of the pooled results. The removal of the influential study Y05 from the "1.2.3 Digital health exercise plus nutrition/health exercise plus education vs health education/offline health exercise plus nutrition" subgroup led to notable changes, as shown in Supplemental Material 2 (b). The effect estimates for this subgroup shifted from a positive to a negative, non-significant mean difference (MD = -0.06, 95% CI [-0.46, 0.34]), and the heterogeneity within this subgroup decreased from $I^2 = 88\%$ to $I^2 = 62\%$, as shown in Supplemental Material 2 (b).

Consequently, the overall pooled effect became smaller and more clearly non-significant (MD = 0.11, 95% CI [-0.07, 0.30], p = 0.24), and the overall heterogeneity was reduced ($I^2 = 77\%$). This analysis confirms that the overall non-significant finding for ASMI is robust. It also identifies study Y05 as a key contributor to the heterogeneity and the positive point estimate in the primary analysis, underscoring that the results for comprehensive digital communication interventions are inconsistent and highly sensitive to the inclusion of specific studies.

(b) Appendicular skeletal muscle mass index - sensitivity analysis

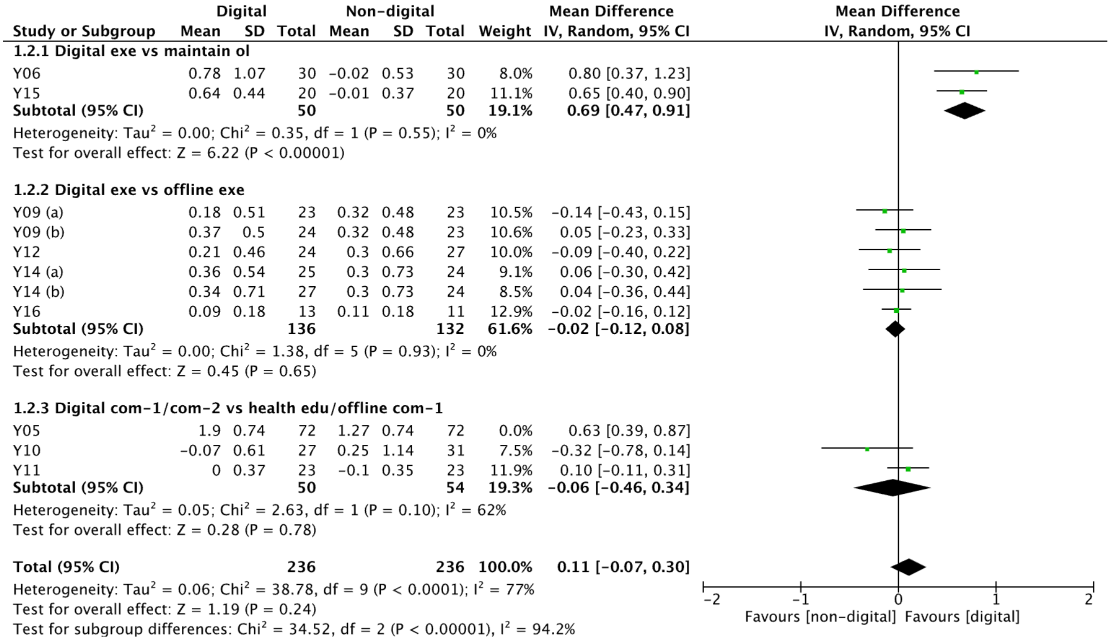

Total skeletal muscle mass

· Effect sizes

The overall meta-analysis, encompassing six pairs of intervention, demonstrated a statistically significant, moderate increase in TSMM favoring digital interventions (Mean Difference = 0.90, 95% CI [0.31, 1.49], $p = 0.003$), as shown in Figure 4 (c).

A clear divergence in effect magnitude was observed between the pre-specified subgroups. In the "1.3.1 Digital exercise vs health education" subgroup, the analysis revealed a small, non-significant effect (MD = 0.23, 95% CI [-0.33, 0.78], $p = 0.42$). Conversely, the "1.3.2 Digital exercise/nutrition/health exercise plus nutrition vs maintain original lifestyle/health education/offline exercise" subgroup showed a large and statistically significant benefit for the more comprehensive digital interventions (MD = 1.25, 95% CI [0.83, 1.67], $p < 0.00001$).

· Heterogeneity analysis

Moderate, borderline significant heterogeneity was detected across the included studies ($I^2 = 54\%$, $Tau^2 = 0.24$, Chi^2 $p = 0.05$). This indicates that a substantial proportion of the variance in observed effects stems from differences between studies rather than sampling error. Notably, heterogeneity within each individual subgroup was negligible ($I^2 = 0\%$), suggesting that the studies within each category are consistent.

· Subgroup analysis

Subgroup analysis was performed to investigate the source of the overall variance. The test for subgroup differences was highly significant ($Chi^2 = 8.26$, $df = 1$, $p = 0.004$, $I^2 = 87.9\%$), confirming that the type of digital intervention and control comparator is a key explanatory factor. The analysis delineates that digital strategies (exercise only, nutrition only, and their combine intervention) are substantially more effective in increasing TSMM than traditional offline interventions.

· Sensitivity analysis

A leave-one-out sensitivity analysis was conducted to evaluate the robustness of the pooled results. The removal of study Y04b, a key study from the "1.3.2 Digital exercise/nutrition/health exercise plus nutrition vs maintain original lifestyle/health education/offline exercise" subgroup, led to observable changes in the overall and subgroup estimates. The overall pooled effect remained statistically significant but

was attenuated, with the mean difference decreasing from 0.90 to 0.73 (95% CI [0.04, 1.41], p = 0.04). The overall heterogeneity was also reduced from $I^2 = 54\%$ to $I^2 = 44\%$. Within this subgroup, the point estimate was slightly lowered but remained large and highly significant (MD = 1.14, 95% CI [0.54, 1.74], p = 0.0002), as shown in Supplemental Material 2 (c).

This analysis confirms that while the magnitude of the overall effect is sensitive to the removal of this particular study, the fundamental conclusion—that comprehensive digital interventions significantly improve TSMM—is robust. The reduction in heterogeneity further suggests that Y04b contributed to the variance in the primary analysis, but its removal does not alter the direction or statistical significance of the key findings.

(c) Total skeletal muscle mass - sensitivity analysis

	Digital			Non-digital				Mean Difference
Study or Subgroup	Mean	SD	Total	Mean	SD	Total	Weight	IV, Random, 95% CI
**1.3.1 Digital exe vs health edu**								
Y01	0.8	3.08	11	−0.4	2.56	12	7.5%	1.20 [−1.13, 3.53]
Y04 (a)	0.07	1.42	50	−0.1	1.51	51	38.8%	0.17 [−0.40, 0.74]
**Subtotal (95% CI)**			**61**			**63**	**46.2%**	**0.23 [−0.33, 0.78]**
Heterogeneity: $Tau^2 = 0.00$; $Chi^2 = 0.71$, df = 1 (P = 0.40); $I^2 = 0\%$								
Test for overall effect: Z = 0.81 (P = 0.42)								
**1.3.2 Digital exe/nut/com-1 vs maintain ol/health edu/offline exe**								
Y04 (b)	1.25	1.47	50	−0.1	1.51	51	0.0%	1.35 [0.77, 1.93]
Y04 (c)	1.09	1.88	50	−0.1	1.51	51	35.4%	1.19 [0.52, 1.86]
Y12	2.13	5.2	24	3.04	6.29	27	4.3%	−0.91 [−4.07, 2.25]
Y15	1.14	2.81	20	−0.21	2.21	20	14.1%	1.35 [−0.22, 2.92]
**Subtotal (95% CI)**			**94**			**98**	**53.8%**	**1.14 [0.54, 1.74]**
Heterogeneity: $Tau^2 = 0.00$; $Chi^2 = 1.71$, df = 2 (P = 0.42); $I^2 = 0\%$								
Test for overall effect: Z = 3.71 (P = 0.0002)								
**Total (95% CI)**			**155**			**161**	**100.0%**	**0.73 [0.04, 1.41]**
Heterogeneity: $Tau^2 = 0.23$; $Chi^2 = 7.16$, df = 4 (P = 0.13); $I^2 = 44\%$								
Test for overall effect: Z = 2.08 (P = 0.04)								
Test for subgroup differences: $Chi^2 = 4.73$, df = 1 (P = 0.03), $I^2 = 78.9\%$								

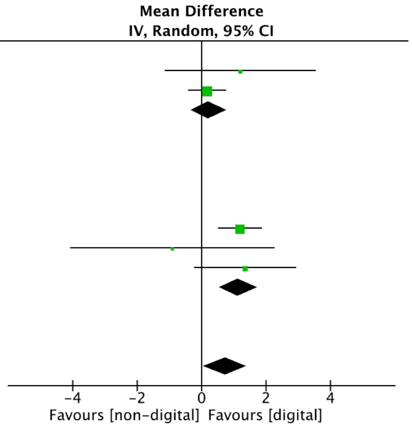

Timed up and go test

· Effect sizes

The overall pooled estimate across nine pairs of intervention demonstrated a negligible and non-significant effect (SMD = -0.02, 95% CI [-0.40, 0.37], p = 0.94), indicating no clear difference in TUGT performance between digital and non-digital groups, as shown in Figure 4 (d).

The effects, however, were inconsistent across the analyzed subgroups. In the "1.4.1 Digital exercise vs offline exercise" subgroup, the effect was also negligible and non-significant (SMD = -0.03, 95% CI [-0.27, 0.21], p = 0.83), suggesting comparable efficacy between digital and traditional offline exercise. In the "1.4.2 Digital exercise/health exercise plus nutrition vs maintain original lifestyle/health education" subgroup, the point estimate was positive but non-significant and displayed very wide confidence intervals (SMD = -0.04, 95% CI [-1.07, 0.99], p = 0.94), reflecting profound inconsistency among the included studies.

· Heterogeneity analysis

Substantial and statistically significant heterogeneity was observed across the studies (I^2 = 80%, Tau^2 = 0.26, Chi^2 $p < 0.00001$). This high level of inconsistency suggests that the true intervention effect may vary significantly. Notably, while the first subgroup was homogeneous (I^2 = 0%), the second subgroup exhibited extreme heterogeneity (I^2 = 94%), which was the primary driver of the overall variance.

· Subgroup analysis

The test for subgroup differences was not statistically significant (Chi^2 = 0.00, df = 1, p = 0.98, I^2 = 0%), indicating that the predefined subgroup classification (based on intervention and control type) did not explain the observed variance in effects. The extreme heterogeneity confined to the second subgroup points to other, unmeasured study characteristics as the source of the inconsistency.

· Sensitivity analysis

A leave-one-out sensitivity analysis was performed by removing study Y02(b), which had a strong, negative effect estimate in the second subgroup. This exclusion markedly altered the results. The effect estimates for the "1.4.2 Digital exercise/health exercise plus nutrition vs maintain original lifestyle/health education " subgroup shifted from a negligible, non-significant effect to a moderate, yet still non-significant, positive

effect (SMD = 0.45, 95% CI [-0.09, 0.99], p = 0.10). Consequently, the overall pooled effect moved in the non-digital direction, though it remained non-significant (SMD = 0.11, 95% CI [-0.17, 0.40], p = 0.44). Most importantly, the overall heterogeneity was substantially reduced from $I^2 = 80\%$ to $I^2 = 52\%$, as shown in Supplemental Material 2 (d).

This sensitivity analysis confirms that the overall null finding is robust. It also identifies study Y02(b) as a critical outlier and a major source of statistical heterogeneity. The removal of this study did not change the non-significant conclusion but provided a more precise and consistent estimate, suggesting that some digital interventions may not show a trend toward benefit, though not conclusively.

(d) Timed up and go test - sensitivity analysis

	Digital			Non-digital				Std. Mean Difference
Study or Subgroup	Mean	SD	Total	Mean	SD	Total	Weight	IV, Random, 95% CI
**1.4.1 Digital exe vs offline exe**								
Y09 (a)	−0.83	0.69	23	−1	0.86	23	12.5%	0.21 [−0.37, 0.79]
Y09 (b)	−0.99	0.8	24	−1	0.86	23	12.7%	0.01 [−0.56, 0.58]
Y12	−0.66	1.31	24	−0.74	1.66	27	13.2%	0.05 [−0.50, 0.60]
Y14 (a)	−0.39	0.7	25	−0.3	0.56	24	12.9%	−0.14 [−0.70, 0.42]
Y14 (b)	−0.58	0.63	27	−0.3	0.56	24	13.0%	−0.46 [−1.02, 0.10]
Y16	−0.54	0.81	13	−0.89	0.81	11	8.4%	0.42 [−0.40, 1.23]
**Subtotal (95% CI)**			**136**			**132**	**72.6%**	**−0.03 [−0.27, 0.21]**
Heterogeneity: $Tau^2 = 0.00$; $Chi^2 = 4.39$, df = 5 (P = 0.49); $I^2 = 0\%$								
Test for overall effect: Z = 0.21 (P = 0.83)								
**1.4.2 Digital exe/com−1 vs maintain ol/health edu**								
Y01	−1.1	4.16	11	−1.2	3.1	12	8.3%	0.03 [−0.79, 0.84]
Y02 (a)	0.5	0.19	64	0.4	0.13	91	19.0%	0.63 [0.30, 0.96]
Y02 (b)	0.3	0.13	68	0.4	0.13	91	0.0%	−0.77 [−1.09, −0.44]
**Subtotal (95% CI)**			**75**			**103**	**27.4%**	**0.45 [−0.09, 0.99]**
Heterogeneity: $Tau^2 = 0.08$; $Chi^2 = 1.81$, df = 1 (P = 0.18); $I^2 = 45\%$								
Test for overall effect: Z = 1.62 (P = 0.10)								
**Total (95% CI)**			**211**			**235**	**100.0%**	**0.11 [−0.17, 0.40]**
Heterogeneity: $Tau^2 = 0.09$; $Chi^2 = 14.62$, df = 7 (P = 0.04); $I^2 = 52\%$								
Test for overall effect: Z = 0.78 (P = 0.44)								
Test for subgroup differences: $Chi^2 = 2.46$, df = 1 (P = 0.12), $I^2 = 59.3\%$								

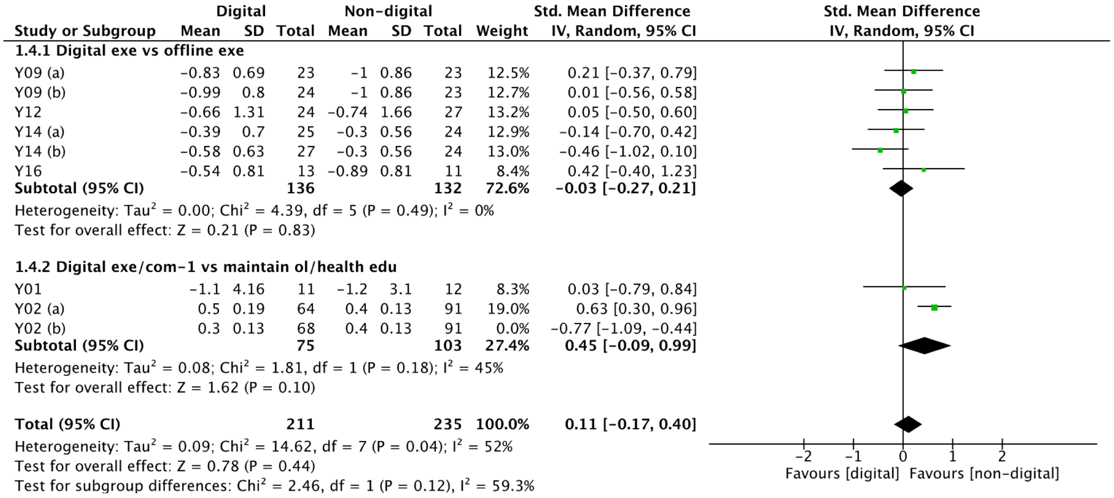

Sit-to-stand test

· Effect sizes

The overall meta-analysis, which included six pairs of intervention, demonstrated a statistically significant, small-to-moderate improvement in STS performance favoring digital interventions, with the effect being precisely at the threshold of significance (Mean Difference = -0.52, 95% CI [-1.04, 0.00], $p = 0.05$), as shown in Figure 4 (e).

A marked divergence in outcomes was evident between the two subgroups. In the "1.5.1 Digital exercise/nutrition/health exercise plus education vs health education" subgroup, the effect was negligible and non-significant (MD = 0.08, 95% CI [-0.56, 0.71], $p = 0.81$). Conversely, the "1.5.2 Digital exercise/health exercise plus nutrition vs maintain original lifestyle/health education" subgroup showed a large and statistically significant benefit for digital interventions (MD = -0.93, 95% CI [-1.31, -0.56], $p < 0.00001$).

· Heterogeneity analysis

Low-to-moderate, non-significant heterogeneity was observed across the studies ($I^2 = 43\%$, $Tau^2 = 0.17$, Chi^2 $p = 0.12$). This suggests that a modest amount of the variance in effect estimates can be attributed to differences between studies. Importantly, heterogeneity within each individual subgroup was negligible ($I^2 = 0\%$ for both), indicating consistency among studies within the same category.

· Subgroup analysis

Subgroup analysis was conducted to explore the differential effects. The test for subgroup differences was statistically significant ($Chi^2 = 7.24$, df = 1, $p = 0.007$, $I^2 = 86.2\%$), confirming that the type of digital intervention is a key factor influencing the outcome. The analysis clearly differentiates between digital strategies that show no clear advantage over health education and those combined digital exercise interventions that yield substantial improvements in physical performance compared to maintenance or education controls.

· Sensitivity analysis

A leave-one-out sensitivity analysis was performed by removing study Y04(b) from the first subgroup. This exclusion led to a notable strengthening of the results. The overall pooled effect became more pronounced and highly significant (MD = -0.71, 95% CI [-1.17, -0.24], $p = 0.003$). Furthermore, the overall heterogeneity was reduced from

I² = 43% to I² = 23%. The effect estimates for the "1.5.1 Digital exercise/nutrition/health exercise plus education vs health education" subgroup shifted slightly towards a negative, non-significant mean difference (MD = -0.04, 95% CI [-0.84, 0.76]), as shown in Supplemental Material 2 (e).

This sensitivity analysis confirms the robustness of the primary finding that certain digital interventions significantly improve STS performance. The removal of Y04(b) not only strengthened the overall effect but also enhanced the precision and consistency of the meta-analysis, underscoring the stability of the conclusion regarding the efficacy of digital interventions (Digital exercise/health exercise plus nutrition).

(e) Sit-to-stand test - sensitivity analysis

	Digital			Non-digital				Mean Difference
Study or Subgroup	Mean	SD	Total	Mean	SD	Total	Weight	IV, Random, 95% CI
**1.5.1 Digital exe/nut/com-2 vs health edu**								
Y04 (a)	-0.15	2.7	50	0.01	2.6	51	16.1%	-0.16 [-1.19, 0.87]
Y04 (b)	0.29	2.75	50	0.01	2.6	51	0.0%	0.28 [-0.76, 1.32]
Y10	-0.97	2.12	27	-1.11	2.77	31	11.6%	0.14 [-1.12, 1.40]
**Subtotal (95% CI)**			**77**			**82**	**27.7%**	**-0.04 [-0.84, 0.76]**
Heterogeneity: Tau² = 0.00; Chi² = 0.13, df = 1 (P = 0.72); I² = 0%								
Test for overall effect: Z = 0.10 (P = 0.92)								
**1.5.2 Digital exe/com-1 vs maintain ol/health edu**								
Y04 (c)	-1.49	3.38	50	0.01	2.6	51	13.0%	-1.50 [-2.68, -0.32]
Y08	-4.4	2.59	40	-3.77	3.69	40	9.7%	-0.63 [-2.03, 0.77]
Y15	-0.89	0.77	20	0	0.54	20	49.5%	-0.89 [-1.30, -0.48]
**Subtotal (95% CI)**			**110**			**111**	**72.3%**	**-0.93 [-1.31, -0.56]**
Heterogeneity: Tau² = 0.00; Chi² = 1.11, df = 2 (P = 0.57); I² = 0%								
Test for overall effect: Z = 4.88 (P < 0.00001)								
**Total (95% CI)**			**187**			**193**	**100.0%**	**-0.71 [-1.17, -0.24]**
Heterogeneity: Tau² = 0.07; Chi² = 5.18, df = 4 (P = 0.27); I² = 23%								
Test for overall effect: Z = 2.99 (P = 0.003)								
Test for subgroup differences: Chi² = 3.93, df = 1 (P = 0.05), I² = 74.6%								

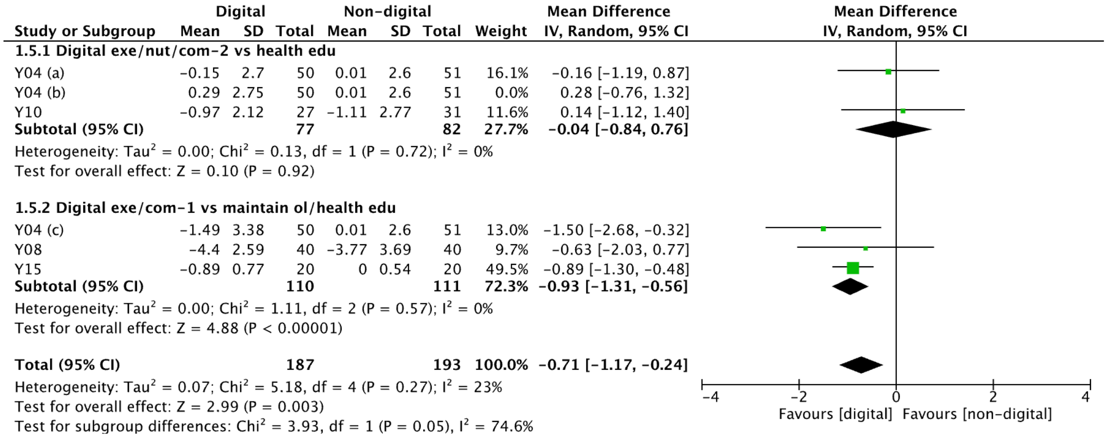

Gait speed

· Effect sizes

The overall meta-analysis, incorporating data from ten pairs of intervention, demonstrated a negligible and non-significant effect on GS (SMD = 0.06, 95% CI [-0.44, 0.56], $p = 0.82$). This indicates no clear difference in gait speed outcomes between the digital and non-digital groups, as shown in Figure 4 (f).

The point estimates were consistently small and non-significant across all three subgroups. The "1.6.1 Digital exercise vs offline exercise" subgroup showed a negligible effect (SMD = 0.06, 95% CI [-0.22, 0.34]). The "1.6.2 Digital exercise/health exercise plus nutrition vs maintain original lifestyle" subgroup suggested a small, non-significant positive effect (SMD = 0.83, 95% CI [-0.73, 2.40]). The "1.6.3 Digital health exercise plus nutrition/health exercise plus education vs health education" subgroup also exhibited a non-significant effect (SMD = -0.65, 95% CI [-1.79, 0.49]).

· Heterogeneity analysis

Substantial and statistically significant heterogeneity was observed across the studies ($I^2 = 84\%$, $Tau^2 = 0.44$, Chi^2 $p < 0.00001$). This high inconsistency suggests that the true effect likely differs significantly across the included studies. Heterogeneity was negligible in the first subgroup ($I^2 = 0\%$) but extreme in the second and third subgroups ($I^2 = 92\%$ and 89%, respectively).

· Subgroup analysis

The test for subgroup differences was not statistically significant ($Chi^2 = 2.39$, $df = 2$, $p = 0.30$, $I^2 = 16.4\%$). This indicates that the predefined categorization based on intervention and control type did not successfully explain the considerable statistical heterogeneity present in the overall analysis. The source of the high variance appears to lie elsewhere, potentially in specific study methodologies or populations.

· Sensitivity analysis

A stepwise leave-one-out sensitivity analysis was performed to identify influential studies and assess the robustness of the null finding, as shown in Supplemental Material 2 (f). Removal of study Y08: Excluding this study from the third subgroup led to a notable reduction in overall heterogeneity, from $I^2 = 84\%$ to $I^2 = 66\%$. The overall effect estimate shifted slightly but remained non-significant (SMD = 0.23, 95% CI [-0.14, 0.60], $p = 0.22$). Removal of studies Y08 and Y15: Further excluding study Y15, a large

outlier from the second subgroup, resulted in a dramatic resolution of heterogeneity. The overall I^2 statistic dropped to 0%. The resulting pooled estimate was a negligible and highly consistent null effect (SMD = 0.04, 95% CI [-0.18, 0.27], p = 0.72).

This comprehensive sensitivity analysis confirms that the overall null finding is robust. It identifies studies Y08 and Y15 as the primary drivers of the extreme statistical heterogeneity in the primary analysis. Once these outliers are accounted for, the data consistently demonstrates no significant effect of digital interventions on gait speed.

(f) Gait speed - sensitivity analysis

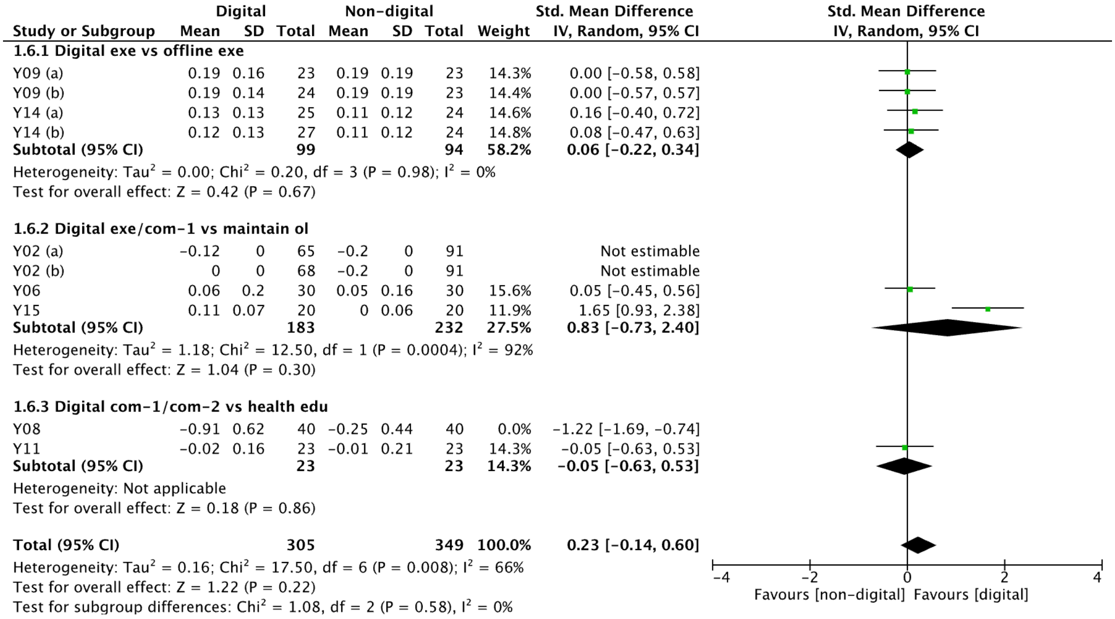

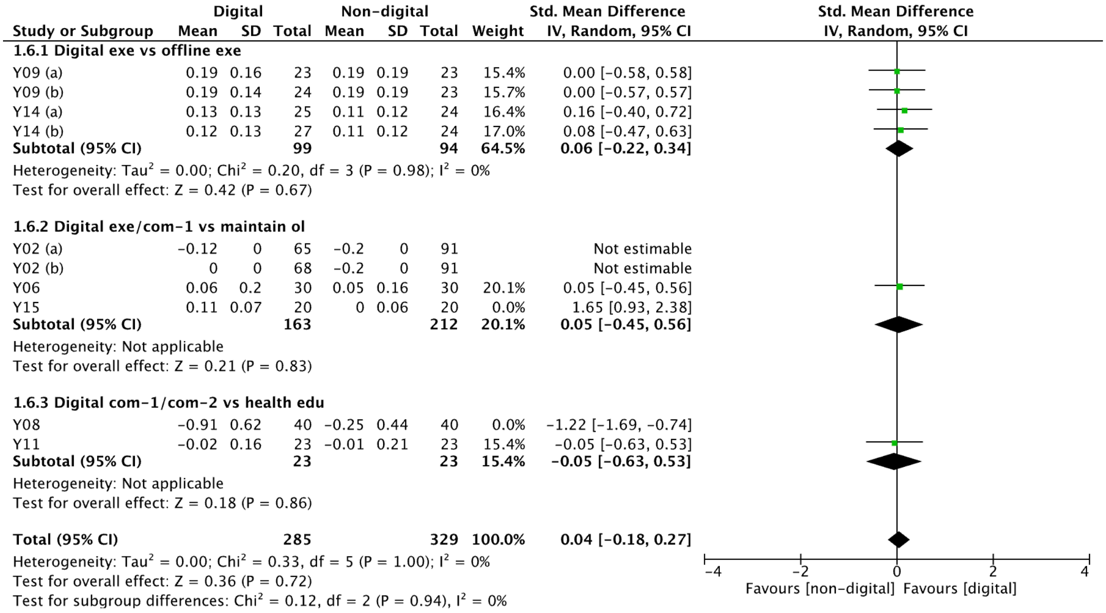

Quality of life

· Effect sizes

The overall meta-analysis, which included seven pairs of intervention, demonstrated a modest but statistically significant improvement in QoL favoring digital interventions (SMD = 0.28, 95% CI [0.04, 0.51], $p = 0.02$), as shown in Figure 4 (g).

The magnitude of this effect, however, was contingent upon the nature of the digital intervention, as evidenced by the subgroup analysis. In the "1.7.1 Digital exercise vs offline exercise" subgroup, the effect was negligible and non-significant, indicating comparable outcomes between digital and traditional offline exercise (SMD = 0.08, 95% CI [-0.18, 0.35], $p = 0.54$). Conversely, the "1.7.2 Digital health exercise plus nutrition/health exercise plus education vs health education" subgroup showed a moderate and statistically significant benefit for comprehensive digital strategies over health education alone (SMD = 0.65, 95% CI [0.29, 1.01], $p = 0.0004$).

· Heterogeneity analysis

Low and non-significant heterogeneity was observed across the included studies ($I^2 = 18\%$, $Tau^2 = 0.02$, Chi^2 $p = 0.29$). This indicates a high level of consistency in the effect estimates, with most of the variability being attributable to chance rather than genuine differences between the studies. Both subgroups also exhibited negligible heterogeneity ($I^2 = 0\%$).

· Subgroup analysis

Subgroup analysis was conducted to explore the differential effects. The test for subgroup differences was statistically significant ($Chi^2 = 6.20$, $df = 1$, $p = 0.01$, $I^2 = 83.9\%$), confirming that the type of intervention is a key factor. This analysis delineates that comprehensive digital programs are effective in enhancing QoL, whereas digital exercise programs alone do not confer a significant advantage over offline exercise.

· Sensitivity analysis

A leave-one-out sensitivity analysis was conducted to evaluate the robustness of the pooled results. The analysis revealed that no single study exerted a disproportionate influence on the overall effect size or heterogeneity. The conclusion of a statistically significant, modest overall benefit remained unchanged regardless of which study was omitted. This confirms that the primary finding is robust and not driven by any particular outlier.

Body mass index

· Effect sizes

The overall pooled estimate across five pairs of intervention demonstrated a negligible and non-significant effect on BMI (Mean Difference = -0.06, 95% CI [-1.28, 1.17], p = 0.93), as shown in Figure 4 (h).

However, a profound and opposing pattern of effects was observed between the two pre-specified subgroups. In the "1.8.1 Digital exercise/health exercise plus education vs maintain original lifestyle/health education" subgroup, a non-significant trend favoring digital interventions for BMI reduction was observed (MD = -0.99, 95% CI [-1.63, -0.35]). In stark contrast, the "1.8.2 Digital nutrition/health exercise plus nutrition vs health education" subgroup demonstrated a significant increase in BMI associated with the digital interventions (MD = 1.33, 95% CI [0.62, 2.03], p = 0.0002).

· Heterogeneity analysis

An extreme level of heterogeneity was observed across the included studies (I^2 = 90%, Tau^2 = 1.72, Chi^2 p < 0.00001). This indicates that the differences in effect sizes between studies are too great to attribute to chance alone, rendering the overall pooled mean difference an unreliable indicator of a single true effect.

· Subgroup analysis

Subgroup analysis was pivotal in explaining the source of the inconsistency. The test for subgroup differences was highly significant (Chi^2 = 22.82, df = 1, p < 0.00001, I^2 = 95.6%), confirming that the type of digital intervention is the principal factor accounting for the heterogeneity. The analysis reveals a critical divergence: digital interventions focused on exercise and exercise plus education for weight loss tend to reduce BMI, whereas digital nutrition and nutrition combined interventions may lead to a significant increase in BMI compared to health education.

· Sensitivity analysis

A leave-one-out sensitivity analysis was performed to assess the stability of the overall null finding and the strong subgroup effects. The analysis confirmed that no single study unduly influenced the overall pooled estimate or the highly significant subgroup differences. The consistent results across these analyses underscore the robustness of the conclusion that the effect of digital interventions on BMI is not uniform and is fundamentally dependent on the intervention's specific components and

objectives.

SUPPLEMENT MATERIAL 4

CERTAINTY OF ECIDENCE

Author(s): Ya Shi
Question: Digital intervention compared to traditional intervention for handgrip strength
Setting: Community, nursing home, hospital-to-home
Bibliography: Shi Y, Stanmore E, McGarrigle L, Todd C. Effectiveness of digital health exercise interventions on muscle function and physical performance in older adults with possible, confirmed or severe sarcopenia: a protocol for a systematic review. BMJ Open. 2024;14(10):e086124. doi:10.1136/bmjopen-2024-086124

Certainty assessment							№ of patients		Effect		Certainty	Importance
№ of studies	Study design	Risk of bias	Inconsistency	Indirectness	Imprecision	Other considerations	digital intervention	traditional intervention	Relative (95% CI)	Absolute (95% CI)		
**Digital exe vs maintain ol**												
3	non-randomised studies	serious[a]	serious[b]	serious[c]	serious[d]	publication bias strongly suspected all plausible residual confounding would suggest spurious effect, while no effect was observed[e]	135	161	-	MD **2.34 kg higher** (0.78 lower to 5.46 higher)	⨁◯◯◯ Very low[a,b,c,d,e]	CRITICAL
**Digital exe vs offline exe**												
6	randomised trials	very serious[f]	not serious	not serious	serious[g]	publication bias strongly suspected all plausible residual confounding would reduce the demonstrated effect[h]	136	132	-	MD **0 kg** (0.58 lower to 0.59 higher)	⨁◯◯◯ Very low[f,g,h]	CRITICAL
**Digital com-2 vs health edu**												
2	non-randomised studies	serious[i]	not serious	serious[j]	serious[k]	publication bias strongly suspected all plausible residual confounding would suggest spurious effect, while no effect was observed[l]	50	54	-	MD **0.24 kg higher** (1.42 lower to 1.9 higher)	⨁◯◯◯ Very low[i,j,k,l]	CRITICAL
**Digital com-1 vs maintain ol/health edu/offline com-1**												
3	randomised trials	serious[m]	serious[n]	serious[o]	not serious	publication bias strongly suspected all plausible residual confounding would reduce the demonstrated effect[p]	180	203	-	MD **2.21 kg higher** (1.33 higher to 3.09 higher)	⨁◯◯◯ Very low[m,n,o,p]	CRITICAL

CI: confidence interval; **MD:** mean difference

Explanations

a. The overall risk of bias was judged as serious for the following reasons. The evidence base consists of three studies. While two studies (Y6 and Y15) were assessed as low risk of bias, one study (Y2) was rated as having 'some concerns' using the RoB 2.0 tool. Given that this study contributes meaningfully to the pooled estimate, the potential for bias in its results introduces a plausible threat to the validity of the overall evidence, warranting downgrading by one level.
b. The evidence was downgraded for serious inconsistency. The statistical heterogeneity is extremely high ($I^2 = 92\%$). Visually, the forest plot demonstrates a clear lack of overlap between the confidence interval of the cluster-RCT (Y2) and the intervals of both the RCT (Y6) and the quasi-experimental study (Y15), despite substantial overlap between the latter two. This large and unexplained variation in effect estimates substantially reduces the confidence in the pooled result.
c. The evidence was downgraded for serious indirectness. Two of the three included studies (Y2 and Y6) enrolled populations that were only partially composed of individuals with sarcopenia, and neither specified the use of a recognized diagnostic guideline for sarcopenia. Consequently, the applicability of the majority of the evidence to a clearly defined sarcopenia population, as specified in the review question, is uncertain.
d. The evidence was downgraded for serious imprecision. The 95% confidence interval around the pooled mean difference is wide (-0.78 kg to 5.46 kg). It includes both the line of no effect (0 kg) and spans from a trivial negative effect to a clinically important positive effect (assuming a minimal important difference of approximately 5 kg for grip strength). This high level of uncertainty renders the result inconclusive for clinical decision-making.
e. The evidence was downgraded one level for other considerations. Publication bias is strongly suspected given the small number of small studies. Furthermore, the inclusion of a quasi-experimental design introduces a high likelihood of residual confounding (e.g., from healthier participant motivation) that would plausibly bias the results toward an overestimation of the intervention effect.
f. The overall risk of bias was judged as very serious. Five out of the six included studies (Y9a, Y9b, Y12, Y14a, Y14b) were assessed as having a high risk of bias, primarily due to concerns in the domain 'bias due to missing outcome data' (D3). As these studies constitute the vast majority of the evidence and contribute substantially to the pooled estimate, the findings are highly likely to be compromised by bias.
g. The evidence was downgraded for serious imprecision. The 95% confidence interval is wide enough to include both trivial harm and trivial benefit (-0.58 kg to 0.59 kg), with a point estimate of no effect (0.00 kg). The total sample size is insufficient to draw a reliable conclusion regarding the effect direction, rendering the result inconclusive.
h. Publication bias is strongly suspected. The number of included studies is small (n=6), the total sample size is limited, and the pooled result shows no effect. These factors increase the likelihood that small, unpublished studies with null or negative findings are missing from the evidence base.
i. The overall risk of bias was judged as serious. One study (Y10) was assessed at high risk of bias due to problems in the randomization process of clusters, which poses a serious threat to the validity of its result. The other study (Y11) raised some concerns across multiple domains. Since half of the evidence comes from a study with a high risk of bias, confidence in the entire body of evidence is substantially reduced.
j. The evidence was downgraded for serious indirectness. One of the two included studies (Y10) enrolled a population that may only partially composed of individuals with sarcopenia. Consequently, the applicability of half of the evidence to a clearly defined sarcopenia population is uncertain, which substantially reduces the confidence in the directness of the overall result.
k. The evidence was downgraded for serious imprecision. The 95% confidence interval is very wide (-1.42 kg to 1.90 kg) and includes the line of no effect (0 kg). With a total sample size of only 104 participants, the result is inconclusive, as the true effect could range from a trivial harm to a trivial benefit.
l. The evidence was downgraded one level for other considerations. Publication bias is strongly suspected due to the very limited evidence base, which consists of only two small studies with a total sample size of 104 participants and a pooled null result, increasing the likelihood that unpublished studies with negative findings are missing. Furthermore, a large effect can be ruled out as the point estimate is trivial (0.24 kg) and the confidence interval excludes the possibility of a large benefit or harm. This decision is further supported by the likelihood of residual confounding, particularly from the quasi-experimental study design, which would plausibly bias the results toward suggesting a spurious positive effect rather than underestimating a true one. Finally, no evidence of a dose-response gradient was reported.
m. The overall risk of bias was judged as serious. All three included studies were assessed with 'some concerns' using the RoB 2.0 tool. The concerns predominantly related to the randomization process (D1), with one study (Y2b) having additional concerns regarding deviations from intended interventions (D2). As the entire body of evidence is affected by these methodological limitations, confidence in the pooled result is substantially reduced.
n. The evidence was downgraded for serious inconsistency. The statistical heterogeneity was extremely high ($I^2 = 94\%$), indicating that the variation between studies is almost entirely due to genuine differences rather than chance. This is supported visually by the forest plot, which shows only partial overlap of the confidence intervals. The substantial and unexplained variability in results substantially reduces the confidence in the pooled estimate.
o. The evidence was downgraded for serious indirectness. Two of the three included studies (Y2b and Y5) raise major concerns regarding the applicability of the population. Y2b enrolled only a partially sarcopenic population, and both Y2b and Y5 failed to specify the use of any recognized diagnostic criteria for sarcopenia. Consequently, the majority of the evidence is of uncertain directness to a well-defined sarcopenia population, which substantially reduces confidence in the applicability of the overall result.
p. The evidence was downgraded for other considerations due to a strong suspicion of publication bias in this small body of evidence (3 studies). Although the precise effect is beneficial, the limited number of studies increases the risk that unpublished null findings are missing. A large effect is absent, no dose-response was seen, and any confounding from the identified methodological concerns would likely underestimate, not create, the observed effect. This does not, however, offset the primary concern regarding publication bias.

Author(s): Ya Shi
Question: Digital intervention compared to traditional intervention for muscle mass
Setting: Community, nursing home, hospital-to-home
Bibliography: Shi Y, Stanmore E, McGarrigle L, Todd C. Effectiveness of digital health exercise interventions on muscle function and physical performance in older adults with possible, confirmed or severe sarcopenia: a protocol for a systematic review. BMJ Open. 2024;14(10):e086124. doi:10.1136/bmjopen-2024-086124

Certainty assessment							№ of patients		Effect		Certainty	Importance
**№ of studies**	**Study design**	**Risk of bias**	**Inconsistency**	**Indirectness**	**Imprecision**	**Other considerations**	**digital intervention**	**traditional intervention**	**Relative (95% CI)**	**Absolute (95% CI)**		
**Digital exe vs maintain ol (appendicular skeletal muscle mass index)**												
2	non-randomised studies	not serious	not serious	serious[a]	not serious	all plausible residual confounding would reduce the demonstrated effect	50	50	-	MD **0.69 kg/m2 higher** (0.47 higher to 0.91 higher)	⊕⊕⊕⊕ High[a]	CRITICAL
**Digital exe vs offline exe (appendicular skeletal muscle mass index)**												
6	randomised trials	very serious[b]	not serious	not serious	serious[c]	publication bias strongly suspected all plausible residual confounding would suggest spurious effect, while no effect was observed[d]	136	132	-	MD **0.02 kg/m2 lower** (0.12 lower to 0.08 higher)	⊕◯◯◯ Very low[b,c,d]	CRITICAL
**Digital com-1/com-2 vs health edu/offline com-1 (appendicular skeletal muscle mass index)**												
3	non-randomised studies	serious[e]	serious[f]	serious[g]	serious[h]	publication bias strongly suspected all plausible residual confounding would suggest spurious effect, while no effect was observed[i]	122	126	-	MD **0.17 kg/m2 higher** (0.31 lower to 0.65 higher)	⊕◯◯◯ Very low[e,f,g,h,i]	CRITICAL
**Digital exe vs health edu (total skeletal muscle mass)**												
2	randomised trials	very serious[j]	not serious	serious[k]	serious[l]	publication bias strongly suspected all plausible residual confounding would suggest spurious effect, while no effect was observed[m]	61	63	-	MD **0.23 kg higher** (0.33 lower to 0.78 higher)	⊕◯◯◯ Very low[j,k,l,m]	CRITICAL
**Digital exe/nut/com-1 vs maintain ol/health edu/offline exe (total skeletal muscle mass)**												
4	non-randomised studies	very serious[n]	not serious	not serious	not serious	publication bias strongly suspected all plausible residual confounding would reduce the demonstrated effect[o]	144	149	-	MD **1.25 kg higher** (0.83 higher to 1.67 higher)	⊕⊕◯◯ Low[n,o]	CRITICAL

CI: confidence interval; **MD:** mean difference

Explanations

a. The evidence was downgraded for serious indirectness. One of the two included studies (Y6) enrolled a population that was only partially composed of individuals with sarcopenia. Consequently, the applicability of half of the evidence to a purely sarcopenic population is uncertain, which reduces the directness and confidence in the overall result.
b. The overall risk of bias was judged as very serious. Five out of the six included studies (Y9a, Y9b, Y12, Y14a, Y14b) were assessed as having a high risk of bias, primarily due to concerns in the domain 'bias due to missing outcome data' (D3). As these studies constitute the vast majority of the evidence and contribute substantially to the pooled estimate, the findings are highly likely to be compromised by bias.
c. The evidence was downgraded for serious imprecision. The 95% confidence interval (-0.12 to 0.08) includes both the line of no effect (0) and spans the range of trivial harm to trivial benefit. Although the interval is relatively narrow, the available sample size is insufficient to determine whether digital exercise is truly equivalent to offline exercise or if it has a small detrimental or beneficial effect on appendicular skeletal muscle mass index. The result is therefore inconclusive for clinical decision-making.
d. The evidence was downgraded one level for other considerations. Publication bias is strongly suspected given the null finding and the possibility of missing small, negative studies. A large effect is absent, and no dose-response gradient was observed. While the studies are rigorous, any residual confounding would be likely to create a spurious effect in either direction around the null, rather than mask a true effect.
e. The overall risk of bias was judged as serious. One study (Y10) was assessed at high risk of bias due to problems in the randomization process of clusters. The other two studies (Y5 and Y11) raised some concerns, primarily in the domains of randomization and confounding. Since the entire body of evidence is affected, and one-third of it comes from a study with a high risk of bias, confidence in the pooled result is substantially reduced.
f. The evidence was downgraded for serious inconsistency. There was considerable heterogeneity in the results, as indicated by an I^2 statistic of 88%. Visually, the forest plot demonstrates a clear lack of overlap, with one study (Y5) showing no overlap with the other two (Y10 and Y11), which themselves had only partial overlap. This substantial unexplained variability substantially reduces the confidence in the pooled estimate.
g. The evidence was downgraded for serious indirectness. Two of the three included studies (Y5 and Y10) raised major concerns regarding the definition of the target population. Y5 did not specify the diagnostic criteria for sarcopenia, and Y10, while mentioning a guideline, did not report the specific inclusion criteria for sarcopenia. Consequently, the applicability of the majority of the evidence to a consistently and well-defined sarcopenia population is uncertain, which substantially reduces confidence in the directness of the overall result.
h. The evidence was downgraded for serious imprecision. The 95% confidence interval is wide (-0.31 to 0.65) and includes the line of no effect (0 kg). The result is inconclusive, as the true effect could range from a trivial negative effect to a moderate positive effect. The sample size is insufficient to provide a precise estimate.
i. The evidence was downgraded one level for other considerations. Publication bias is strongly suspected given the limited number of small studies and the null finding. A large effect is absent, and no dose-response gradient was observed. Furthermore, the serious risk of bias in the evidence base means that any plausible residual confounding would be likely to create a spurious effect around the null, rather than underestimate a true effect.
j. The overall risk of bias was judged as very serious. Both included studies (Y1 and Y4a) were assessed as having a high risk of bias. The concerns in Y4a related to the randomization process at both the cluster and individual levels, and both studies had serious issues with missing outcome data. As the entire body of evidence originates from studies with a high risk of bias, the confidence in the pooled result is very low.
k. The evidence was downgraded for serious indirectness. One of the two included studies (Y1) did not use an internationally recognized diagnostic guideline for sarcopenia and, crucially, did not confirm that its population consisted solely of individuals with sarcopenia. This creates uncertainty regarding the applicability of half of the evidence to the well-defined target population of interest, reducing the directness of the overall result.
l. The evidence was downgraded for serious imprecision. The 95% confidence interval is wide (-0.33 to 0.78) and includes the line of no effect (0). With a total sample size of only 124 participants, the result is inconclusive, as the true effect could range from a trivial negative effect to a moderate positive effect.
m. The evidence was downgraded one level for other considerations. Publication bias is strongly suspected given the very limited number of small, high-risk-of-bias studies and the imprecise null finding. A large effect is absent, and no dose-response gradient

was observed. Furthermore, the high risk of bias in both studies means that any plausible residual confounding would be likely to create a spurious effect around the null.
n. The overall risk of bias was judged as very serious. Three of the four included studies (Y4b, Y4c, Y12) were assessed as having a high risk of bias. The concerns in Y4b and Y4c related to critical flaws in the randomization process at both the cluster and individual levels, as well as serious issues with missing outcome data. Y12 also had a high risk of bias due to missing outcome data. As the vast majority of the evidence originates from studies with a high risk of bias, the confidence in the pooled result is very low.
o. The evidence was downgraded one level for other considerations due to a strong suspicion of publication bias in this small body of evidence where most studies have a high risk of bias. A large effect is not present, and no dose-response gradient was observed. The identified methodological flaws in the majority of studies would, if anything, likely lead to an underestimation of the true effect.

Author(s): Ya Shi
Question: Digital intervention compared to traditional intervention for physical performance
Setting: Community, nursing home, hospital-to-home
Bibliography: Shi Y, Stanmore E, McGarrigle L, Todd C. Effectiveness of digital health exercise interventions on muscle function and physical performance in older adults with possible, confirmed or severe sarcopenia: a protocol for a systematic review. BMJ Open. 2024;14(10):e086124. doi:10.1136/bmjopen-2024-086124

Certainty assessment							№ of patients		Effect		Certainty	Importance
№ of studies	Study design	Risk of bias	Inconsistency	Indirectness	Imprecision	Other considerations	digital intervention	traditional intervention	Relative (95% CI)	Absolute (95% CI)		
**Digital exe vs offline exe (timed up and go test)**												
6	randomised trials	very serious[a]	not serious	not serious	serious[b]	publication bias strongly suspected all plausible residual confounding would suggest spurious effect, while no effect was observed[c]	136	132	-	SMD **0.03 SD lower** (0.27 lower to 0.21 higher)	⨁◯◯◯ Very low[a,b,c]	CRITICAL
**Digital exe/com-1 vs maintain ol/health edu (timed up and go test)**												
3	randomised trials	serious[d]	very serious[e]	serious[f]	serious[g]	publication bias strongly suspected all plausible residual confounding would suggest spurious effect, while no effect was observed[h]	143	194	-	SMD **0.04 SD lower** (1.07 lower to 0.99 higher)	⨁◯◯◯ Very low[d,e,f,g,h]	CRITICAL
**Digital exe/nut/com-2 vs health edu (sit-to-stand test)**												
3	randomised trials	very serious[i]	not serious	serious[j]	serious[k]	publication bias strongly suspected all plausible residual confounding would suggest spurious effect, while no effect was observed[l]	127	133	-	MD **0.08 s higher** (0.56 lower to 0.71 higher)	⨁◯◯◯ Very low[i,j,k,l]	CRITICAL
**Digital exe/com-1 vs maintain ol/health edu (sit-to-stand test)**												
3	non-randomised studies	serious[m]	not serious	not serious	not serious	publication bias strongly suspected all plausible residual confounding would reduce the demonstrated effect[n]	110	111	-	MD **0.93 s lower** (1.31 lower to 0.56 lower)	⨁⨁⨁◯ Moderate[m,n]	CRITICAL
**Digital exe vs offline exe (gait speed)**												
4	randomised trials	very serious[o]	not serious	not serious	serious[p]	publication bias strongly suspected all plausible residual confounding would suggest spurious effect, while no effect was observed[q]	99	94	-	SMD **0.06 SD higher** (0.22 lower to 0.34 higher)	⨁◯◯◯ Very low[o,p,q]	CRITICAL
**Digital exe/com-1 vs maintain ol (gait speed)**												
4	non-randomised studies	not serious	very serious[r]	serious[s]	serious[t]	publication bias strongly suspected all plausible residual confounding would suggest spurious effect, while no effect was observed[u]	183	232	-	SMD **0.83 SD higher** (0.73 lower to 2.4 higher)	⨁◯◯◯ Very low[r,s,t,u]	CRITICAL
**Digital com-1/com-2 vs health edu (gait speed)**												
2	non-randomised studies	not serious	serious[v]	not serious	serious[w]	publication bias strongly suspected all plausible residual confounding would suggest spurious effect, while no effect was observed[x]	63	63	-	SMD **0.65 SD lower** (1.79 lower to 0.49 higher)	⨁⨁◯◯ Low[v,w,x]	CRITICAL

CI: confidence interval; **MD:** mean difference; **SMD:** standardised mean difference

Explanations

a. The overall risk of bias was judged as very serious. Five of the six included studies (Y9a, Y9b, Y12, Y14a, Y14b) were assessed as having a high risk of bias due to concerns regarding missing outcome data (Domain 3). As the vast majority (83%) of the evidence originates from studies with a high risk of bias, the confidence in the pooled result is very low.
b. The evidence was downgraded for serious imprecision. The 95% confidence interval (-0.27 to 0.21) includes both the line of no effect (0) and spans from a small negative effect to a small positive effect. Although the total sample size appears moderate, the result is inconclusive as it cannot rule out the possibility that digital exercise is either slightly inferior or slightly superior to offline exercise. The available sample size is insufficient to draw a reliable conclusion regarding the comparative effectiveness of the two interventions.
c. The evidence was downgraded one level for other considerations. Publication bias is strongly suspected given the imprecise null finding in a body of evidence composed mostly of small, high-risk-of-bias studies. A large effect is absent, and no dose-response gradient was observed. Furthermore, the prevailing bias from missing data could plausibly create a spurious effect in either direction around the null.
d. The overall risk of bias was judged as serious. One study (Y1) was assessed at high risk of bias due to missing outcome data. The other two studies (Y2a and Y2b) raised some concerns regarding the randomization process and deviations from intended interventions. As the entire body of evidence is affected by these limitations, and one-third of it comes from a study with a high risk of bias, confidence in the pooled result is substantially reduced.
e. The evidence was downgraded two levels for very serious inconsistency. The statistical heterogeneity was extremely high ($I^2 = 94\%$). Visually, the forest plot demonstrates profound inconsistency: the confidence intervals of studies Y2a and Y2b show no overlap and lie on opposite sides of the line of no effect, indicating diametrically opposed results. The remaining study (Y1) also shows very limited overlap with the others. This large, unexplained variability renders the pooled estimate uninterpretable.
f. The evidence was downgraded for serious indirectness. All three included studies raise major concerns regarding the applicability of the population. Study Y1 used a non-standard, investigator-defined diagnostic criteria for sarcopenia. Studies Y2a and Y2b did not report any diagnostic criteria and enrolled only partially sarcopenic populations. Consequently, the entire body of evidence is of uncertain directness to a well-defined sarcopenia population, which substantially reduces confidence in the applicability of the overall result.
g. The evidence was downgraded for serious imprecision. The 95% confidence interval is very wide (-1.07 to 0.99 seconds) and includes both the line of no effect (0) and values representing a clinically important improvement and a clinically important harm. The result is inconclusive for clinical decision-making, as the true effect could be either beneficial or harmful to a meaningful degree.
h. The evidence was downgraded one level for other considerations. Publication bias is strongly suspected given the limited number of studies, profound inconsistency, and imprecise null finding. A large effect is absent, and no dose-response gradient was observed. The methodological weaknesses and heterogeneity further suggest that any confounding would likely create spurious effects rather than mask a true effect.
i. The overall risk of bias was judged as very serious. All three included studies (Y4a, Y4b, and Y10) were assessed as having a high risk of bias. The concerns predominantly related to critical flaws in the randomization process of clusters (and individuals for Y4a/Y4b). As the entire body of evidence (100%) originates from studies with a high risk of bias, the confidence in the pooled result is very low.
j. The evidence was downgraded for serious indirectness. One of the three included studies (Y10), while focused on sarcopenia prevention, did not confirm the sarcopenia status of its participants. This creates uncertainty regarding the applicability of a portion of the evidence to a confirmed sarcopenia population, which reduces the directness of the overall result.
k. The evidence was downgraded for serious imprecision. The 95% confidence interval is wide (-0.56 to 0.71 seconds) and includes the line of no effect (0). The result is inconclusive, as the true effect could range from a meaningful improvement to a meaningful worsening in sit-to-stand time. The available sample size is insufficient to provide a reliable estimate of the effect direction or magnitude.
l. The evidence was downgraded one level for other considerations. Publication bias is strongly suspected given the limited number of small, high-risk-of-bias studies and the imprecise null finding. A large effect is absent, and no dose-response gradient was observed. Furthermore, the high risk of bias across all studies means that any plausible residual confounding would be likely to create a spurious effect around the null.
m. The overall risk of bias was judged as serious. One study (Y4c) was assessed at high risk of bias due to critical flaws in the randomization process and missing outcome data. Another study (Y8) raised some concerns regarding the randomization process. As the majority of the evidence (two out of three studies) is affected by these methodological limitations, confidence in the pooled result is substantially reduced.
n. The evidence was downgraded one level for other considerations due to a strong suspicion of publication bias in this small body of evidence. A large effect is not present, and no dose-response gradient was observed. The direction of the potential bias from the methodological flaws suggests that the true effect might be larger than observed, but this does not offset the concern regarding publication bias.
o. The overall risk of bias was judged as very serious. All four included studies (Y9a, Y9b, Y14a, Y14b) were assessed as having a high risk of bias due to concerns regarding missing outcome data (Domain 3). As the entire body of evidence (100%) originates from studies with a high risk of bias, the confidence in the pooled result is very low.
p. The evidence was downgraded for serious imprecision. The 95% confidence interval is wide (-0.22 to 0.34 m/s) and includes the line of no effect (0). The result is inconclusive for clinical decision-making, as the true effect could range from a clinically important harm to a clinically important benefit. The available sample size is insufficient to provide a precise estimate.
q. The evidence was downgraded one level for other considerations. Publication bias is strongly suspected given the very limited number of small, high-risk-of-bias studies and the imprecise null finding. A large effect is absent, and no dose-response gradient was observed. Furthermore, the high risk of bias from missing data across all studies means that any plausible residual confounding would be likely to create a spurious effect around the null.
r. The evidence was downgraded two levels for very serious inconsistency. The statistical heterogeneity was extremely high ($I^2 = 92\%$). Furthermore, the results are uninterpretable: two studies (Y2a, Y2b) reported standard deviations of zero, making their effect sizes not estimable and their data unreliable. The remaining two studies (Y6, Y15) show completely non-overlapping confidence intervals, indicating diametrically opposed results. This combination of unreliable data and contradictory findings renders any pooled estimate meaningless.
s. The evidence was downgraded for serious indirectness. Three of the four included studies (Y2a, Y2b, Y6) did not use a recognized diagnostic criteria for sarcopenia and enrolled only partially sarcopenic populations. Consequently, the majority of the evidence is of uncertain directness to a well-defined sarcopenia population, which substantially reduces confidence in the applicability of the overall result.
t. The evidence was downgraded for serious imprecision. The 95% confidence interval is extremely wide (-0.73 to 2.40) and includes the line of no effect (0). The result is inconclusive for clinical decision-making, as the true effect could represent anything from a substantial harm to a very large benefit. The available sample size is insufficient to provide a reliable estimate of the effect direction or magnitude.
u. The evidence was downgraded one level for other considerations. Publication bias is strongly suspected given the limited number of studies, profound inconsistency, and highly imprecise result. A large effect is absent, and no dose-response gradient was observed. The severe methodological limitations (inconsistency, indirectness) suggest that any confounding would be likely to create or exaggerate spurious effects.
v. The evidence was downgraded for serious inconsistency. There was considerable heterogeneity in the results, as indicated by an I^2 statistic of 89%. Visually, the forest plot demonstrates a clear lack of overlap between the confidence intervals of the two studies. This large and unexplained variability in effect estimates substantially reduces the confidence in the pooled result.
w. The evidence was downgraded for serious imprecision. The 95% confidence interval is extremely wide (-1.79 to 0.49 m/s) and includes the line of no effect (0). With a very small total sample size (N=126), the result is inconclusive, as the true effect could represent anything from a large harmful effect to a moderate beneficial effect.
x. The evidence was downgraded one level for other considerations. Publication bias is strongly suspected given the very limited number of small studies, high inconsistency, and an imprecise null finding. A large effect is absent, and no dose-response gradient was observed. The methodological concerns in both studies further suggest that any residual confounding would be likely to create spurious effects around the null.

Author(s): Ya Shi
Question: Digital intervention compared to traditional intervention for other indexes
Setting: Community, nursing home, hospital-to-home
Bibliography: Shi Y, Stanmore E, McGarrigle L, Todd C. Effectiveness of digital health exercise interventions on muscle function and physical performance in older adults with possible, confirmed or severe sarcopenia: a protocol for a systematic review. BMJ Open. 2024;14(10):e086124. doi:10.1136/bmjopen-2024-086124

Certainty assessment							№ of patients		Effect		Certainty	Importance
№ of studies	Study design	Risk of bias	Inconsistency	Indirectness	Imprecision	Other considerations	digital intervention	traditional intervention	Relative (95% CI)	Absolute (95% CI)		
**Digital exe vs offline exe (quality of life)**												
5	randomised trials	very serious[a]	not serious	not serious	serious[b]	publication bias strongly suspected all plausible residual confounding would suggest spurious effect, while no effect was observed[c]	114	109	-	SMD **0.08 SD higher** (0.18 lower to 0.35 higher)	⊕◯◯◯ Very low[a,b,c]	CRITICAL
**Digital com-1/com-2 vs health edu (quality of life)**												
2	non-randomised studies	not serious	not serious	not serious	not serious	publication bias strongly suspected all plausible residual confounding would reduce the demonstrated effect[d]	63	63	-	SMD **0.65 SD higher** (0.29 higher to 1.01 higher)	⊕⊕⊕⊕ High[d]	CRITICAL
**Digital exe/com-2 vs maintain ol/health edu (body mass index)**												
3	non-randomised studies	serious[e]	not serious	serious[f]	not serious	publication bias strongly suspected all plausible residual confounding would reduce the demonstrated effect[g]	97	102	-	MD **0.99 kg/m2 lower** (1.63 lower to 0.35 lower)	⊕⊕◯◯ Low[e,f,g]	CRITICAL
**Digital nut/com-1 vs health edu (body mass index)**												
2	randomised trials	very serious[h]	not serious	not serious	not serious	publication bias strongly suspected all plausible residual confounding would suggest spurious effect, while no effect was observed[i]	100	102	-	MD **1.33 kg/m2 higher** (0.62 higher to 2.03 higher)	⊕⊕◯◯ Low[h,i]	CRITICAL

CI: confidence interval; **MD:** mean difference; **SMD:** standardised mean difference

Explanations

a. The overall risk of bias was judged as very serious. Four of the five included studies (Y9a, Y9b, Y14a, Y14b) were assessed as having a high risk of bias due to concerns regarding missing outcome data (Domain 3). The remaining study (Y13) raised some concerns. As the vast majority (80%) of the evidence originates from studies with a high risk of bias, and given the particular susceptibility of patient-reported outcomes like quality of life to bias from missing data, confidence in the pooled result is very low.
b. The evidence was downgraded for serious imprecision. The 95% confidence interval is wide enough to include both the line of no effect (0) and the threshold for a minimal important difference (assuming an MID of approximately 0.2 to 0.5 in the scale units). With a total sample size of 223 participants, the result is inconclusive, as the true effect could range from a trivial negative effect to a positive effect of potential importance.
c. The evidence was downgraded one level for other considerations. Publication bias is strongly suspected given the limited number of small, high-risk-of-bias studies and the imprecise null finding. A large effect is absent, and no dose-response gradient was observed. Furthermore, the high risk of bias from missing data across most studies suggests that any residual confounding would be likely to create a spurious effect around the null.
d. The evidence was downgraded one level for other considerations due to a strong suspicion of publication bias in this very limited body of evidence. A large effect is not present, and no dose-response gradient was observed. The direction of the potential bias from the methodological concerns suggests that the true effect might be larger than observed, but this does not offset the primary concern regarding publication bias.
e. The overall risk of bias was judged as serious. Two of the three included studies (Y4a and Y10) were assessed at high risk of bias, with concerns predominantly in the critical domain of the randomization process for clusters. As the majority of the evidence originates from studies with a high risk of bias, confidence in the pooled result is substantially reduced.
f. The evidence was downgraded for serious indirectness. One of the three included studies (Y10) did not specify the diagnostic criteria or confirm the sarcopenia status of its population. This creates uncertainty regarding the applicability of a portion of the evidence to the well-defined sarcopenia population of interest, which reduces the directness of the overall result.
g. The evidence was downgraded one level for other considerations due to a strong suspicion of publication bias in this limited body of evidence. A large effect is not present, and no dose-response gradient was observed. The direction of the potential bias from the methodological flaws in the majority of studies suggests that the true effect might be larger than observed, but this does not offset the concern regarding publication bias.
h. The overall risk of bias was judged as very serious. Both included studies (Y4b and Y4c) were assessed as having a high risk of bias. The concerns related to critical flaws in the randomization process at both the cluster and individual levels, as well as serious issues with missing outcome data. As the entire body of evidence (100%) originates from studies with a high risk of bias, the confidence in the pooled result is very low.
i. The evidence was downgraded one level for other considerations. Publication bias is strongly suspected given the very limited number of small, high-risk-of-bias studies. A large effect is absent, and no dose-response gradient was observed. Furthermore, the high risk of bias in both studies means that any plausible residual confounding would be likely to create or exaggerate a spurious effect, particularly given the direction of the observed outcome.
